# A chemical chaperone improves muscle function in mice with a RyR1 mutation

**DOI:** 10.1038/ncomms14659

**Published:** 2017-03-24

**Authors:** Chang Seok Lee, Amy D. Hanna, Hui Wang, Adan Dagnino-Acosta, Aditya D. Joshi, Mark Knoblauch, Yan Xia, Dimitra K. Georgiou, Jianjun Xu, Cheng Long, Hisayuki Amano, Corey Reynolds, Keke Dong, John C. Martin, William R. Lagor, George G. Rodney, Ergun Sahin, Caroline Sewry, Susan L. Hamilton

**Affiliations:** 1 Department of Molecular Physiology and Biophysics, Baylor College of Medicine, One Baylor Plaza, Houston, Texas 77030, USA; 2 Dubowitz Neuromuscular Centre, UCL Institute of Child Health and Great Ormond Street Hospital, 30 Guilford Street, London WC1N 1EH, UK

## Abstract

Mutations in the *RYR1* gene cause severe myopathies. Mice with an I4895T mutation in the type 1 ryanodine receptor/Ca^2+^ release channel (RyR1) display muscle weakness and atrophy, but the underlying mechanisms are unclear. Here we show that the I4895T mutation in RyR1 decreases the amplitude of the sarcoplasmic reticulum (SR) Ca^2+^ transient, resting cytosolic Ca^2+^ levels, muscle triadin content and calsequestrin (CSQ) localization to the junctional SR, and increases endoplasmic reticulum (ER) stress/unfolded protein response (UPR) and mitochondrial ROS production. Treatment of mice carrying the I4895T mutation with a chemical chaperone, sodium 4-phenylbutyrate (4PBA), reduces ER stress/UPR and improves muscle function, but does not restore SR Ca^2+^ transients in I4895T fibres to wild type levels, suggesting that decreased SR Ca^2+^ release is not the major driver of the myopathy. These findings suggest that 4PBA, an FDA-approved drug, has potential as a therapeutic intervention for RyR1 myopathies that are associated with ER stress.

## Abstract (web summary)

Mutations in the RyR1 channel cause core myopathies. Here the authors show that ER stress and the unfolded protein response underlie the pathology caused by a common RyR1 channel mutation, and show that treatment with a chemical chaperone restores muscle function in mice.

Core myopathies are the most common form of congenital myopathies and more than half of these myopathies arise from mutations in the gene (*RYR1*) for the skeletal muscle Ca^2+^ release channel or type 1 ryanodine receptor (RyR1)^1^. *RYR1* mutations in the human population occur at frequencies as high as 1 in 2000 (ref. 2). In mice some mutations in the *Ryr1* gene increase SR Ca^2+^ leak^3^ while others decrease Ca^2+^ permeation through RyR1 (ref. 4), raising the question of how mutations with opposing effects on both the amplitude of the Ca^2+^ transient and resting cytosolic Ca^2+^ concentrations produce similar disease symptoms. We have shown that a mutation in *Ryr1* that leads to the replacement of tyrosine with a serine at amino acid 524 in RyR1 (Y522S in humans, Y524S in mice), which cause both malignant hyperthermia and myopathy with cores, increases heat sensitivity, temperature-dependent SR Ca^2+^ leak, mitochondrial damage and oxidative/nitrosative stress^5^. Despite substantial progress towards developing interventions for myopathies arising from Ca^2+^ leak in RyR1 (ref. 6), there are currently no FDA-approved drugs available for any RyR1 myopathy. The heat-induced life-threatening response in transgenic mice carrying the Y524S mutation is prevented by treatment with 5-aminoimidazole-4-carboxamide ribonucleoside^7^ and the myopathy is alleviated by anti-oxidants^5^. Since, as described in this manuscript, the IT mutation decreases RyR1 Ca^2+^ permeation and Ca^2+^ leak, it is unlikely that interventions that further decrease Ca^2+^ leak via RyR1 will be beneficial (and may even be harmful) in patients with mutations that either decrease Ca^2+^ permeation through RyR1 or affect its interaction with SR-lumenal proteins.

To develop interventions for RyR1 myopathies arising from RyR1 mutations that do not produce Ca^2+^ leak, we created mice with the I4895T mutation (equivalent to I4898T in humans). This mutation, identified initially by Lynch *et al*.^8^, is one of the most common RyR1 mutations in humans and has a variable presentation and penetrance ranging from myopathies such as Central Core Disease, which can present extremely mild to severe muscle impairment in a single family^9^,^10^,^11^ to a lethal early onset congenital core-rod myopathy^12^. Mice with the I4895T mutation on a different genetic background^13^ display a highly variable phenotype ranging from a severe muscle phenotype that dramatically increases in severity with age to a mild, non-progressive myopathy. The ultrastructural changes detected in these mice were found to be consistent with premature muscle ageing^14^. However, for the most part, RyR1 myopathies are early onset and non-progressive or slowly progressive^15^ suggesting that the subset of I4895T mice with the severe, age-dependent phenotype are not truly representative of human early onset, slowly progressive RyR1 myopathy. In the present study we demonstrate that persistent endoplasmic reticulum (ER) stress/UPR and excessive mitochondrial reactive oxygen species (ROS) production contribute to muscle weakness and atrophy in mice with the I4895T mutation in RyR1 and that both the weakness and the atrophy are reversed by the treatment of these IT mice with a chemical chaperone, 4-phenylbutyrate (4PBA).

## Results

### Generation and phenotyping of IT mice

We generated mice with the I4895T mutation in RyR1 as described in Supplementary Fig. 1. These mice were backcrossed >10 generations onto a C57B6/J background. The I4895T mutation was homozygous lethal and, therefore, all experiments were performed with the heterozygous I4895T male mice (designated IT) and wild type (WT) littermates. Parallel studies with female mice are ongoing. When an adequate number of WT littermates were not available we used carefully aged matched WT mice from our colony on a C57B6/J background.

**Figure 1:**
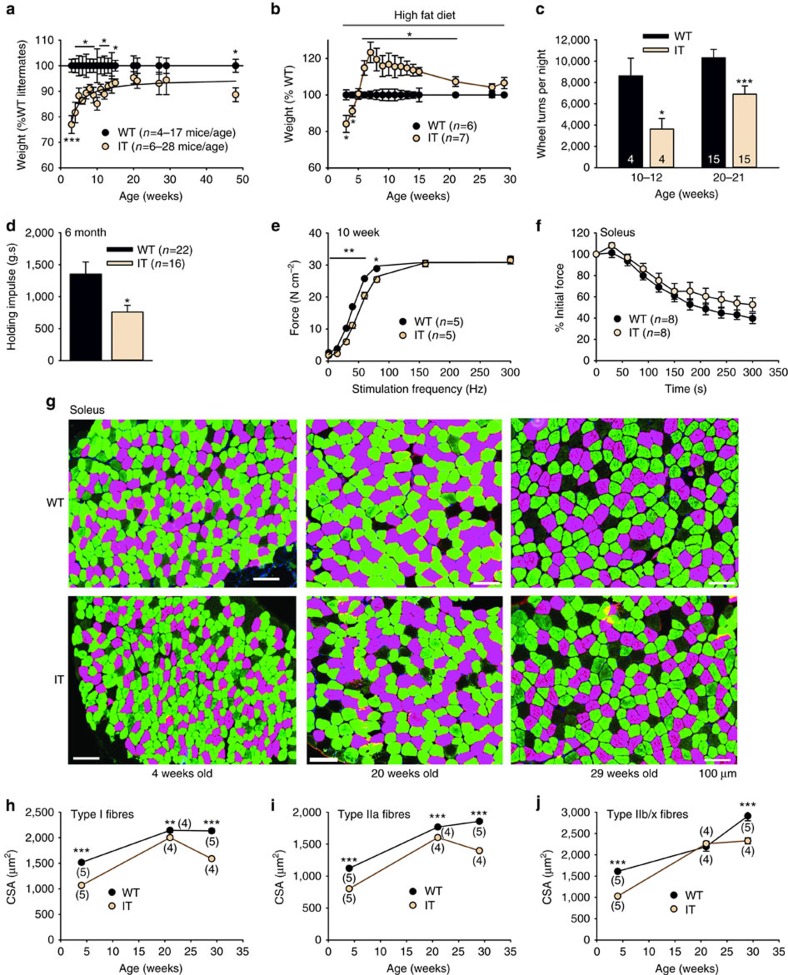
Phenotype of I4898T mice. (**a**) Body weight of IT and WT littermates on a normal chow diet as a function of age. (**b**) Body weight of mice on a high fat diet as a function of age. (**c**) Monitored running in WT and IT mice at two ages. (**d**) Holding impulse (Body weight (g) × the maximal length of time that mice (29 weeks) hang from a wire without falling. (**e**) Force frequency for the soleus muscle of 10-week-old WT and IT mice. (**f**) Fatigue in the soleus of 10-week-old WT and IT mice (plotted as % initial force) with 15 Hz stimulation train. (**g**) Representative fibre type immunostaining images in soleus muscles of IT and WT at different ages using specific myosin heavy chain antibodies. Figures shown are pseudo-coloured magenta for type I fibres, green for type IIa and black for type IIb/x. Scale bars are 100 μm. (**h**–**j**) Analysis of fibre CSA of different fibre types in the soleus of IT and WT. Distributions of CSAs for these muscles are shown in Supplementary Fig. 5. *n* is the number of mice used in each experiment. Data are shown as mean±s.e.m. *P<0.05, **P<0.01, ***P<0.001. For statistical analysis we used a two-tailed Student's *t*-test, with differences considered significant at *P*<0.05.

**Figure 2:**
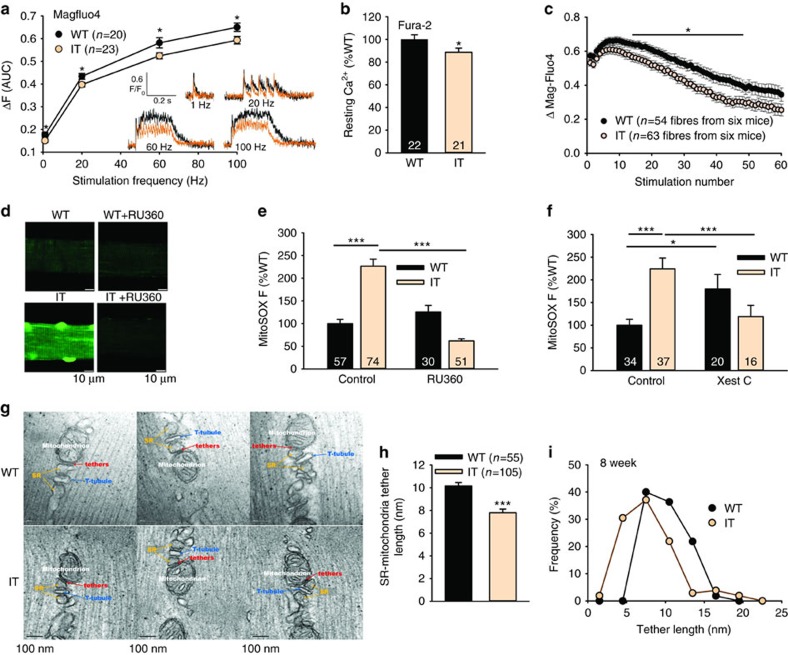
Ca^2+^ handling. (**a**) Analysis of Ca^2+^ release in FDB fibres (area under the curve, AUC) at different stimulation frequencies using Magfluo4. Inset: Representative Ca^2+^ transients at different stimulation frequencies. *n* is the number of fibres from at least three different mice. (**b**) Resting cytosolic Ca^2+^ concentration relative to that of WT fibres assessed with Fura-2 in FDB fibres. *n* is the number of fibres from at least three different mice. (**c**) Analysis of the amplitudes of the Ca^2+^ transients in FDB fibres from WT and IT mice with repetitive 100 Hz (200 ms train/1.5 s) stimulations. (**d**) Representative fibres loaded with MitoSOX from WT and IT mice with and without RU360 treatment. Scale bars are 10 μm. (**e**) Analysis of images in panel D. MitoSOX fluorescence in FDB fibres (with and without RU360 to inhibit MCU) from WT and IT mice was plotted as % WT control (littermate, same day). *n* is the number of fibres from three to four mice. (**f**) Effect of Xestospongin C on MitoSox fluorescence. (**g**) Negatively stained EM pictures of SR-MaMs from IT and WT soleus. Bar is 100 nm. (**h**) Analysis of the average length of the tethers between the SR and the interfibrillar mitochondria. (**i**) Distribution of tether length in WT and IT soleus. Data are shown as mean±s.e.m. *P<0.05, **P<0.01, ***P<0.001 (two-tailed Student's *t*-test).

**Figure 3:**
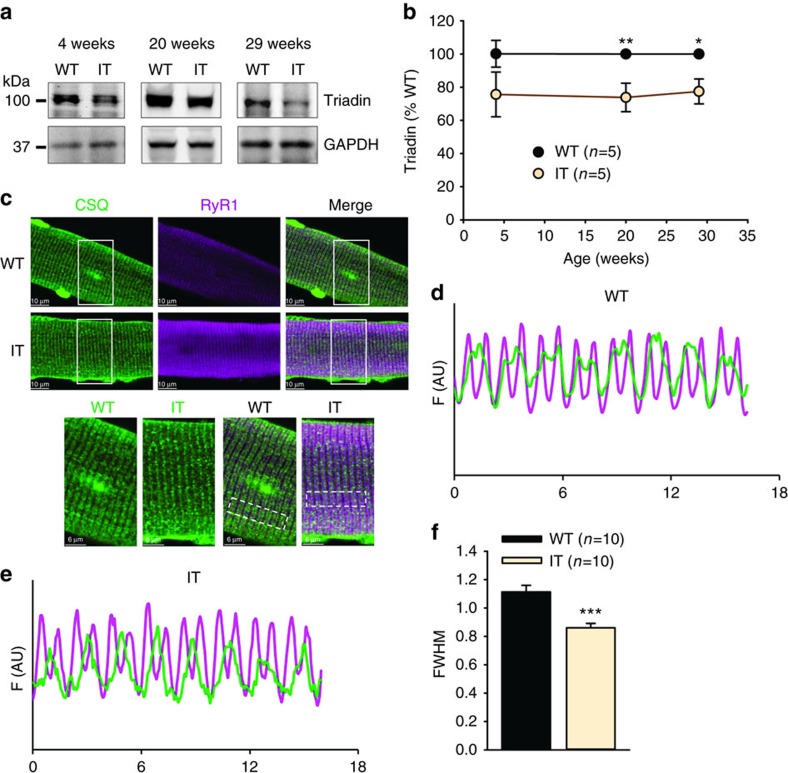
Effect of the IT mutation on triadin and CSQ. (**a**) Representative western blots of triadin levels in soleus muscle from WT and IT mice at different ages. (**b**) Analysis of triadin levels. (**c**) RyR1 and CSQ localization in FDB fibres by immunocytochemistry. Magenta: RyR1, Green: CSQ. (**d**,**e**) Line scans of CSQ and RyR1 immunostaining in FDB fibres of IT (**e**) and WT (**d**) mice. (**f**) Analysis of the fluorescence profile of CSQ in WT and IT fibres, FWHM is full width at half maximum. Data are shown as mean±s.e.m. *P<0.05, **P<0.01, ***P<0.001(two-tailed Student's *t*-test).

**Figure 4:**
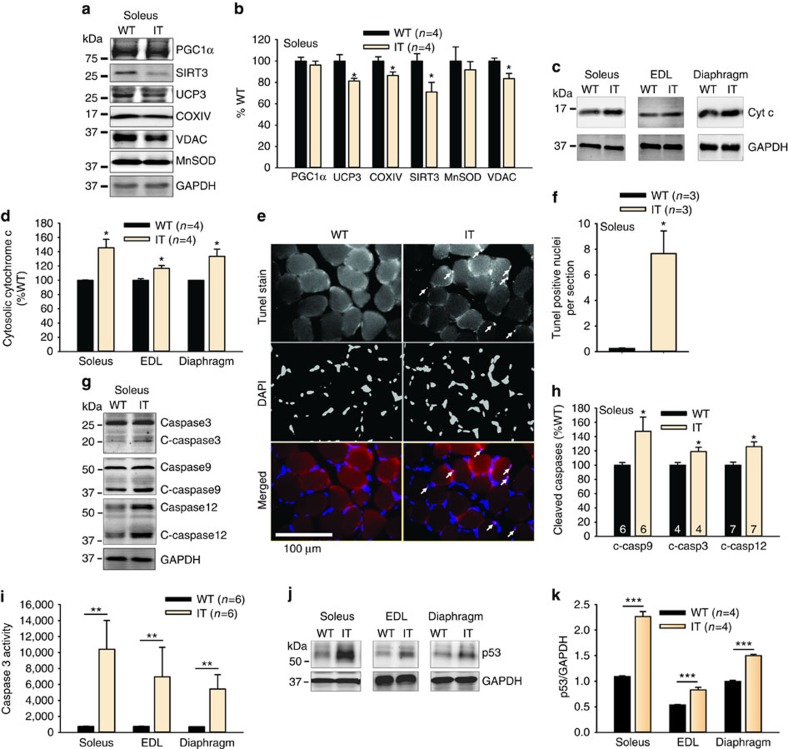
Mitochondrial damage and activation of proapoptotic pathways in the muscle of IT mice. (**a**) Representative western blots for mitochondrial proteins in homogenates of soleus muscles from WT and IT mice. (**b**) Analysis of changes in mitochondrial proteins in homogenates of the soleus muscle of IT compared to WT littermate mice. (**c**) Representative western blots of cytosolic cytochrome c in soleus from IT and WT mice. (**d**) Analysis of cytosolic cytochrome c in the soleus of WT and IT mice. (**e**) TUNEL staining for apoptotic nuclei. Second row of the panel stained for nuclei with DAPI. Third row of the panel is the merged image. (**f**) Analysis of the number of TUNEL positive nuclei per section. (**g**) Representative western blots for caspases. (**h**) Analysis of cleaved caspases. (**i**) Assay for caspase 3 activity. (**j**) Representative western blot for p53 in muscles of IT and WT littermates. (**k**) Analysis of p53 levels in soleus, EDL and diaphragm muscles of WT and IT muscle. *n* is the number of mice used in each experiment. Data are shown as mean±s.e.m. *P<0.05, **P<0.01, ***P<0.001 (two-tailed Student's *t*-test). GAPDH loading controls are below each set of blots from a single gel.

**Figure 5:**
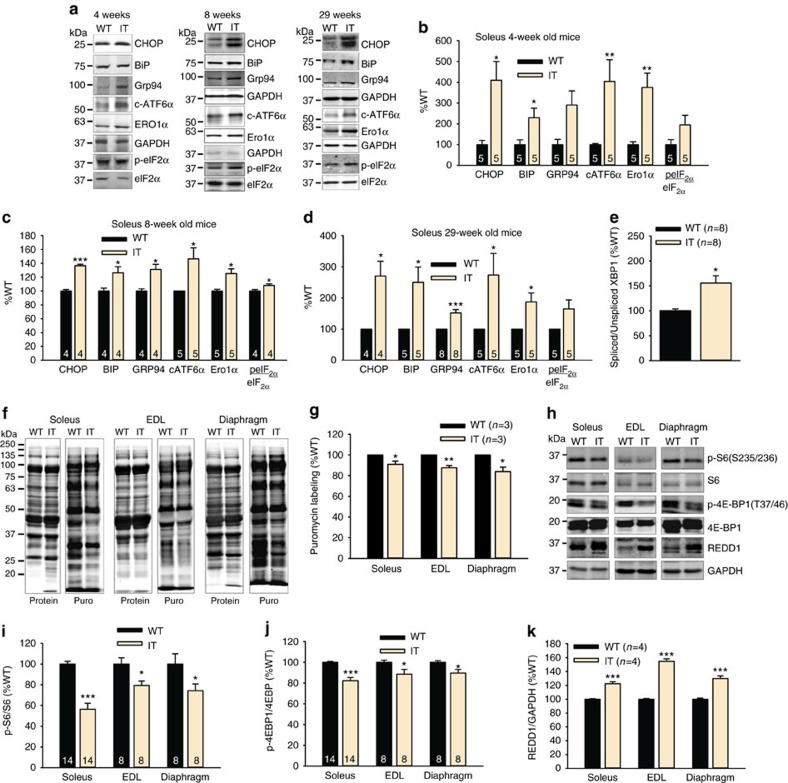
Evidence of ER stress in IT muscle. (**a**) Representative western blot of proteins involved in ER stress in the soleus of 4, 8 and 29-week-old IT and WT mice. (**b**) Analysis of ER stress markers in soleus muscle of 4-week-old IT compared to WT littermate mice. (**c**) Analysis of ER stress markers in soleus muscle of 8-week-old IT compared to WT littermate mice. (**d**) Analysis of ER stress markers in soleus muscle of 29-week-old IT compared to WT littermate mice. (**e**) Analysis of XBP1 splicing by qRT-PCR. (**f**) Representative western blots for newly synthesized puromycin labelled proteins using anti-puromycin antibody for SUnSET analysis. (**g**) Analysis of protein synthesis by SUnSET. (**h**) Representative western blot of proteins downstream of mTORC1 activation and REDD1. (**i**–**k**) Analysis of changes in proteins downstream of mTORC1 activation and REDD1 in soleus, EDL and diaphragm muscle of IT compared to WT mice. *n* is the number of mice used in each experiment. Data are shown as mean±s.e.m. *P<0.05, **P<0.01, ***P<0.001 (two-tailed Student's *t*-test).

**Figure 6:**
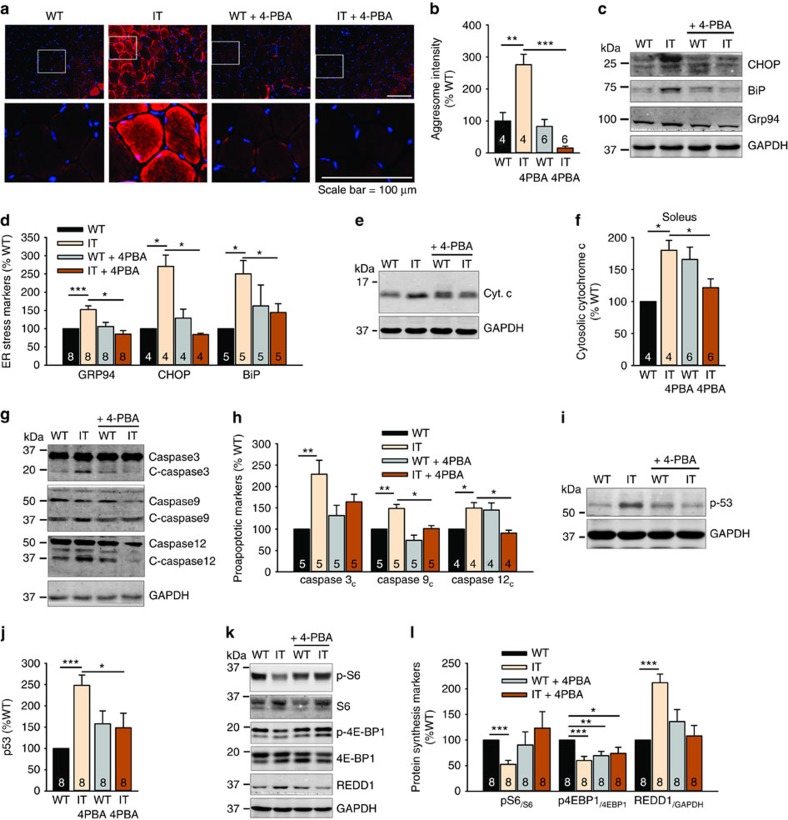
4PBA reverses the signalling changes in IT muscle. (**a**) ProteoStat aggresome staining in the soleus of IT and WT littermates treated for 2 weeks with or without 4PBA. Lower panels are enlarged images boxed in upper panels. Scale bar is 100 μm. (**b**) Analysis of aggresome intensity in the soleus with or without treatment of 4PBA for 2 weeks. (**c**) Representative western blots on ER stress markers from the soleus of WT and IT mice treated for 2 weeks with 4PBA. (**d**) Effects of 4PBA on ER stress markers. (**e**) Representative western blot for cytosolic cytochrome c in the cytosolic fraction of soleus from WT and IT mice treated with and without 4PBA for 2–3 weeks. (**f**) Analysis of cytosolic cytochrome c in muscle from IT and WT littermate mice treated with and without 4PBA. (**g**) Representative western blot showing the effect of 2-week treatment of 4PBA on cleaved caspases in the soleus. (**h**) Effects of 4PBA on cleaved caspases in the soleus. (**i**) Representative western blots of p53 in the soleus of IT and WT littermates without or with 4PBA treatment for 2–3 weeks. (**j**) The effects of 4PBA on p53 levels in soleus. (**k**) Representative western blot of proteins downstream of mTORC1 in the soleus of IT and WT littermates±4PBA treatment for 2–3 weeks. (**l**) The effects of 4PBA on proteins downstream of mTORC1 from >3 pairs of IT and WT littermates. Data are shown as mean±s.e.m. *P<0.05, **P<0.01, ***P<0.001 (two-tailed Student's *t*-test).

**Figure 7:**
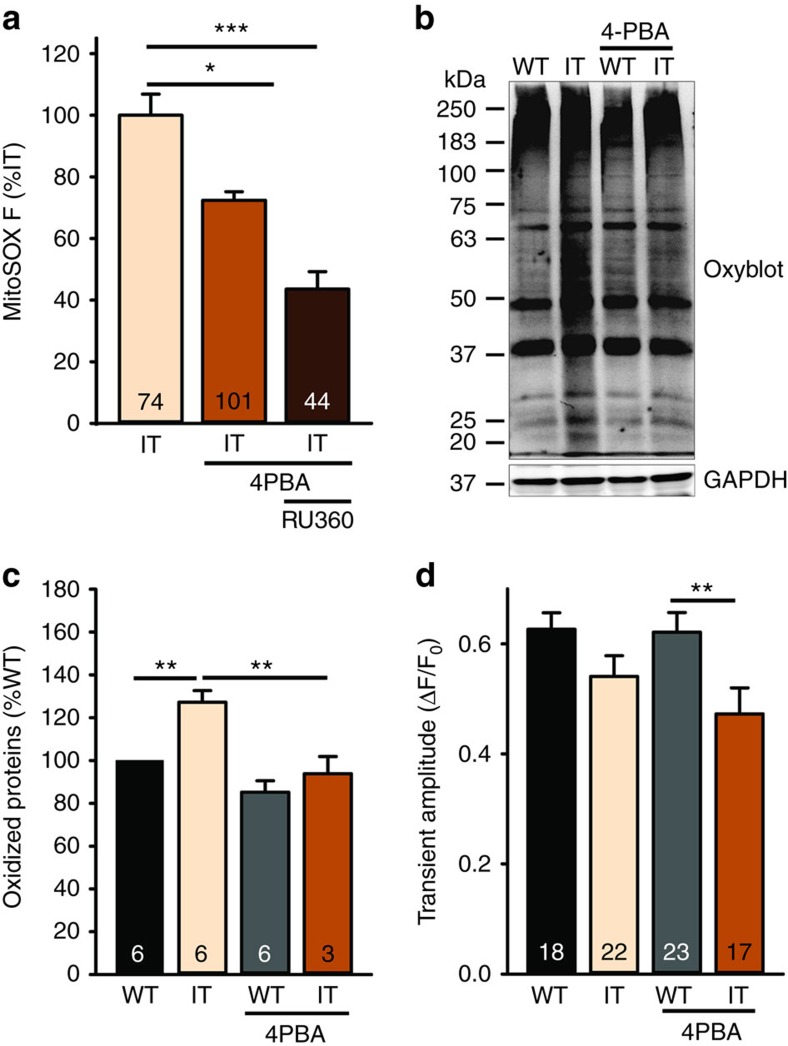
Effect of 4PBA on mitochondrial ROS and RyR1-mediated Ca^2+^ release. (**a**) Analysis of MitoSOX fluorescence in IT treated with and without 4PBA for 3 weeks. Plotted as % IT untreated. Also, shown is the effect of RU360; the number of fibres is in the bar. (**b**) Oxyblot of soleus homogenates from mice treated with or without 4PBA. (**c**) Analysis of oxidized proteins in soleus of mice treated with and without 4PBA. (**d**) Analysis of the effects of 4PBA on the amplitude of the Ca^2+^ transient assessed with Magfluo4. In all panels except A, *n* is the number of mice used in each experiment. Data are shown as mean±s.e.m. *P<0.05, **P<0.01, ***P<0.001 (two-tailed Student's *t*-test).

**Figure 8:**
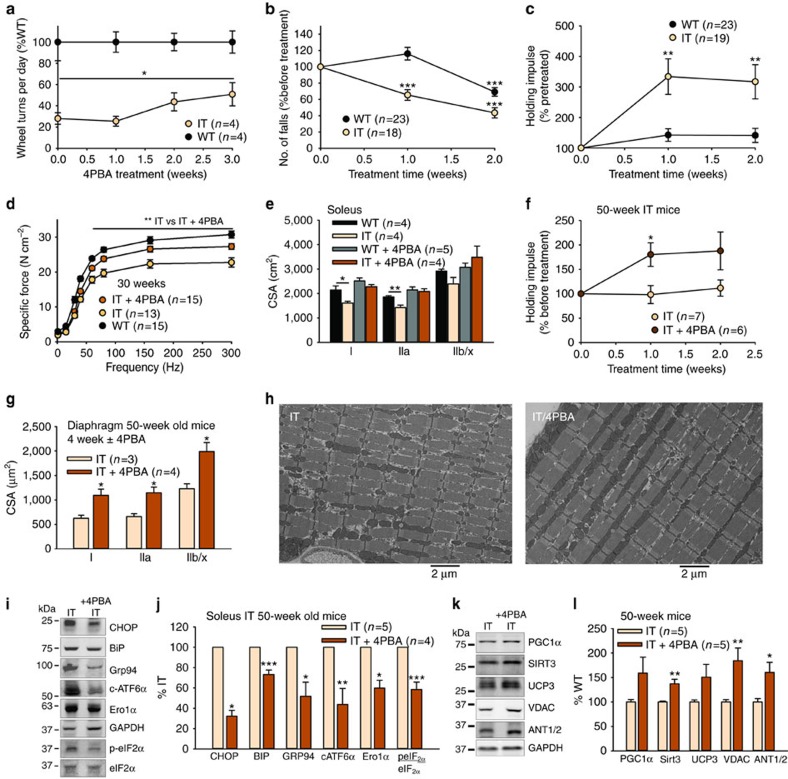
4PBA improves muscle CSA and function in IT mice. (**a**) Wheel turns (%WT) in 10–12-week-old IT and WT mice treated with 4 PBA. (**b**) Number of falls (% before treatment) in a wire hang test (29-week-old mice). Performance is normalized to performance before receiving 4PBA. Significance is assessed by comparing performance after 4PBA with performance before 4PBA. (**c**) Holding impulse (%before treatment, holding time × body weight) for 29-week-old WT and IT mice. (**d**) Force frequency for WT, IT and IT mice treated with 4PBA for 2–3 weeks (age 25–35 weeks). (**e**) CSA of the soleus muscle fibres from 29-week-old mice WT and IT mice±chronic 4PBA. (**f**) Holding impulse of 50-week-old IT and WT mice±4PBA (% pretreatment). (**g**) Diaphragm fibre CSA in 50-week-old IT mice treated±4PBA for 4–6 weeks. (**h**) Negatively stained transmission electron microscopy images of soleus muscle sections from 50-week-old IT mice treated±4PBA. Scale bars are 2 μm. (**i**) Representative western blot of ER stress proteins in 50-week-old IT mice treated±4PBA. (**j**) Analysis of the effects of 4PBA on ER stress markers (plotted as % untreated IT). (**k**) Western blot of mitochondrial proteins from 50-week-old IT mice±4PBA treatment. (**l**) Relative changes in mitochondrial protein content in muscle of 50-week-old IT mice±4PBA treatment. *n* is the number of mice used in each experiment. Data are shown as mean±s.e.m. *P<0.05, **P<0.01, ***P<0.001 (two-tailed Student's *t*-test) For the data in panels A, B, C and F, performance was compared to performance before 4PBA and used a paired *t*-test.

IT mice on a normal chow diet weighed less than their WT littermates with the greatest difference manifesting at young ages (Fig. 1a). However, after 6 weeks on a high fat diet, the IT mice gained more weight (Fig. 1b) than WT mice. IT mice ran significantly less on monitored exercise wheels (Fig. 1c) and performed poorly in wire hang tests compared to WT littermate controls (Fig. 1d). We also assessed muscle-specific (soleus, extensor digitorum longus (EDL) and diaphragm) changes in function. For clarity, we present primarily soleus data in Figs 1, 2, 3, 4, 5, 6, 7, 8. Similar results were obtained in EDL and diaphragm muscles and these data are shown in Supplementary Figs 2 and 3, respectively. In younger mice the force-frequency curves were shifted to the right (∼9 Hz, 10-week-old mice, Fig. 1e) without a major change in maximal specific force production. In older IT mice (30 weeks of age) maximal force was decreased (see Fig. 8d). However, fatigue was not significantly altered by the IT mutation (Fig. 1f).

While the soleus from mice with the IT mutation displayed slightly more IIb/x fibres (Supplementary Fig. 4a–c), we found a significant decrease in the cross-sectional area (CSA) of all fibre types at three different ages (Fig. 1g–j). The frequency distributions of fibres with different CSAs from soleus, EDL and diaphragm muscles of IT and WT mice at different ages are shown in Supplementary Fig. 5.

### Ca^2+^ handling and oxidative stress

Loy *et al*.^4^ found that the IT mutation reduced Ca^2+^ permeation through RyR1 without changing SR lumenal Ca^2+^ levels. We evaluated the effects of the IT mutation on Ca^2+^ handling in isolated flexor digitorum brevis (FDB) fibres using the low affinity Ca^2+^ sensor, Mag-Fluo 4. We found a small reduction in the Ca^2+^ transients at all stimulation frequencies in fibres from IT compared to WT littermate mice (Fig. 2a). The resting cytosolic Ca^2+^ levels (measured with the high affinity Ca^2+^ sensor, Fura-2) were lower in FDB fibres from IT compared to WT littermate mice (Fig. 2b), suggesting that this mutation does not lead to Ca^2+^ leak. However, the relative difference in the amplitudes of the Ca^2+^ transients in IT and WT fibres increased with repetitive, fatiguing stimulations (100 Hz), suggesting the possibility of activity-dependent modifications of RyR1 activity (Fig. 2c).

ER-mitochondrial-associated membranes (MaMs) are sites of Ca^2+^, phospholipid and ROS exchange between the ER and mitochondria^16^,^17^. Similar structures designated SR mitochondrial-associated membranes (SR-MaMs) are found in skeletal muscle^18^ and are thought to play a role in matching contraction-associated energy demands with ATP production via Ca^2+^ signalling to mitochondria^19^. Decreased resting cytosolic Ca^2+^ levels and reduced Ca^2+^ transient amplitudes would be expected to decrease mitochondrial Ca^2+^ uptake in IT compared to WT fibres. To determine if this is indeed the case, we attempted to assess the effects of the IT mutation on Ca^2+^ uptake by mitochondria at the SR-MaMs using Rhod2 (refs 20, 21). However, any changes in passive Ca^2+^ uptake into mitochondria in IT and WT fibres were below the detection limits of Rhod2. Since even small increases in mitochondrial Ca^2+^ uptake are associated with increased mitochondrial ROS production^22^,^23^, we used the mitochondrial ROS sensor, MitoSOX^24^; RU360, an inhibitor of the mitochondrial Ca^2+^ uniporter (MCU); and Xestospongin C, an inhibitor of inositol 1,4,5-trisphosphate receptors^25^ (IP3R), to determine if mitochondrial ROS production was different in WT and IT FDB fibres. The interfibrillar mitochondria in the FDB fibres of the IT mice displayed markedly increased ROS production and this increase in ROS in the IT fibres was blocked by RU360 (Fig. 2d,e) and Xestospongin C (Fig. 2f). Since resting cytosolic Ca^2+^ levels were decreased and mitochondrial ROS production was blocked by both RU360 and Xestospongin C in IT fibres, the increased ROS production is likely to be occurring as the result of increased mitochondrial Ca^2+^ uptake at ER/SR-MaMs. The lack of an effect of RU360 and Xestospongin C in WT fibres suggests that, under normal conditions in WT fibres, Ca^2+^ release to mitochondria at the ER/SR-MaMs is minimal. The absence of a major contribution of mitochondrial Ca^2+^ uptake via the microdomain at the ER/SR MaMs in WT skeletal muscle is consistent with the finding that skeletal muscle interfibrillar mitochondria primarily take up Ca^2+^ in response to large global myoplasmic Ca^2+^ changes during repetitive stimulation^26^,^27^,^28^. Our data suggest that persistent ER stress drives Ca^2+^ uptake and ROS production by mitochondria at the ER/SR-MaMs. IP3Rs are the Ca^2+^ release channels enriched in ER-MaMs^29^ in other cell types and our finding of blockage of mitochondrial ROS production by Xestospongin C suggests that IP3Rs are also the Ca^2+^ release channels at the ER/SR-MaMs in skeletal muscle. The IT mutation appears to be increasing Ca^2+^ uptake, leading to increased ROS production by mitochondria at the ER/SR-MaMs despite decreases in both resting bulk cytosolic Ca^2+^ and voltage gated Ca^2+^ release. The IT mutation in RyR1 did not greatly alter the overall structure of the SR-MaMs (Fig. 2g), but the tethers between the SR and mitochondria were significantly shorter at the IT SR-MaMs (Fig. 2h,i). The decreased distance from the SR to the mitochondria could contribute to the increased mitochondrial Ca^2+^ uptake in IT muscle; however, further analyses of the structure and function of the ER/SR MaMs in IT muscle is needed.

We also assessed the expression levels of Ca^2+^ handling proteins in the IT mice. We found that the levels of Ca^2+^ handling proteins (RyR1, Ca_V_1.1, SERCA2, calsequestrin (CSQ)) in the EDL, diaphragm and soleus were, for the most part, not significantly different in muscles of WT and IT mice (Supplementary Figs 2f,g; 3f,g; 4d,e), but, as expected from the small fibre type shifts, there was a small increase in SERCA1 in the diaphragm (Supplementary Fig. 3g). The IT mutation in RyR1 is located in the selectivity filter but is close to the lumenal binding sites for triadin^30^,^31^. Triadin is required to maintain CSQ at the junctional SR in close proximity to RyR1 (ref. 32) and to optimize excitation–contraction coupling^30^,^33^. We found a decrease in the amount of triadin in IT muscle (Fig. 3a,b) at three different age groups (4, 20 and 29 weeks old) and an alteration in the localization of CSQ to the junctional SR (Fig. 3c–f). Our data suggest that the IT mutation is altering the interactions of RyR1 with proteins in the SR lumen.

### Consequences of persistent Ca^2+^ uptake into the mitochondria

Persistent elevations in mitochondrial Ca^2+^ uptake and ROS production at the ER/SR-MaMs would be expected to cause mitochondrial damage. Consistent with increased mitochondrial damage, mitochondrial protein content was decreased (20-week-old mice, Fig. 4a,b; Supplementary Figs 2h,i; 3h,i) and cytosolic cytochrome c was increased in IT muscle (Fig. 4c,d), suggesting that there has been mitochondrial permeability transition pore opening (mPTP).

Excessive mitochondrial Ca^2+^ uptake at the ER-MaMs also drives the activation of proapoptotic pathways^34^,^35^,^36^. In support of this, we found: (a) increased apoptotic nuclei (Fig. 4e,f), (b) increased cleaved caspases 3, 9 and 12 (Fig. 4g,h), (c) increased caspase 3 activity (Fig. 4i) and (d) marked elevation of the tumour suppressor and proapoptotic protein, p53, compared to WT littermates (Fig. 4j,k). These findings suggest that the persistent increases in mitochondrial Ca^2+^ and ROS production cause both mitochondrial damage and elevation of proapoptotic pathways, thereby contributing to the muscle dysfunction.

### The IT mutation causes ER stress

The above findings raise the question of what drives increased mitochondrial Ca^2+^ uptake/ROS production at the ER/SR–MaMs, leading to damaged mitochondria. The SR is an extensive, specialized domain of the ER with a continuous intralumenal space^37^. Physiological stressors^38^ such as oxidative stress, glucose deprivation, inflammatory cytokines, free fatty acids, and other factors that regulate ER function, can cause the accumulation of unfolded/misfolded proteins that trigger an unfolded protein response (UPR) and ER stress. ER stress/UPR activated signalling, in turn, decreases protein synthesis, upregulates molecular chaperones to facilitate protein folding, and strengthens the contact between the ER and mitochondria to alleviate ER stress, increase ATP production and promote survival^39^. However, persistent or unresolved ER stress further increases mitochondrial Ca^2+^ uptake^34^ and ROS production, induces mPTP, and drives apoptosis^40^. Both ablation of triadin and a mutation in CSQ that leads to its mislocalization are associated with ER stress in cardiac muscle^41^,^42^. The decreased triadin, CSQ mislocalization, increased mitochondrial ROS production, and elevation of proapoptotic markers suggested that the muscle of IT might be displaying persistent ER stress/UPR and this was further explored.

We found that proteins associated with ER stress were significantly elevated in muscles (Fig. 5a–d) of IT compared to WT littermate mice at all ages tested. ER stress-mediated UPR is orchestrated by the action of three signalling proteins, IRE1α (inositol requiring enzyme 1), PERK (protein kinase RNA (PKR)-like ER kinase) and ATF6 (activating transcription factor 6α)^43^. Cleaved ATF6α (the active form of this transcription factor) and the downstream target of ATF6α, CHOP (transcriptional factor C/EBP homologous protein), were both elevated. eIF2A (eukaryotic translation initiation factor 2α) is the downstream target of PERK and the ratio of p-eIF2α to eIF2α was increased in IT muscle. We also analysed the alternative splicing (regulated by IRE1α) of XBP1 (X box-binding protein 1, a transcriptional activator for many of the UPR target genes), and found an increase in XBP1 splicing in the soleus muscle (Fig. 5e). These findings suggest that all three arms of the ER stress/UPR response are persistently elevated in the muscle of the IT mice.

Persistent ER stress decreases protein synthesis, causes mitochondrial damage and activates proapoptotic pathways^44^. As discussed above, both mitochondrial damage and activation of proapoptotic pathways are occurring in IT muscle (Fig. 4). Protein synthesis, assessed with the SUnSET puromycin technique^45^,^46^, was decreased in the muscles of IT compared to WT mice (Fig. 5f,g). Both p-S6/S6 (ribosomal protein S6) and p-4EBP/4EBP1 (eukaroyotic translation iniitation factor 4E-binding protein), markers of mTORC1 activation, were decreased while REDD1 (a negative regulator of mTORC1 activity) was increased in IT muscle (Fig. 5h–k). Decreased protein synthesis is, therefore, likely to contribute to decreased muscle fibre size in the IT mice.

### Mitigation of ER stress with a chemical chaperone

If ER stress is a major contributor to the myopathy associated with the IT mutation, then interventions that alleviate ER stress should normalize the altered pathways and improve muscle function. Chemical chaperones aid in protein folding and reduce protein aggregation and oxidative stress (reviewed in ref. 47). Since ER stress, mitochondrial damage and apoptosis are defining features of IT muscle pathology, we sought to determine if restoration of protein folding with a chemical chaperone, 4PBA, would reverse muscle pathology through reduction of ER stress/UPR and oxidative stress. The data shown in Figs 6 and 7 were obtained with mice treated for >2 weeks with 4PBA in the drinking water. We found that protein aggregates (stained with ProteoStat) (Fig. 6a,b), ER stress markers (Fig. 6c,d), cytosolic cytochrome c (Fig. 6e,f), cleaved caspases (Fig. 6g,h), p53 (Fig. 6i,j), all elevated in IT muscle, were markedly reduced by treating the IT mice with 4PBA. Markers of mTORC1 activation, reduced by the IT mutation, were restored to WT levels by 4PBA (Fig. 6k,l). Treatment with 4PBA also reduced muscle protein ubiquitination (Supplementary Fig. 6a,b) and autophagy markers (Supplementary Fig. 6c,d). Collectively, these findings suggest that alleviating ER stress/UPR normalizes the pathways that are altered by the IT mutation.

We next addressed the critical question of whether 4PBA affects ROS production by the interfibrillar mitochondria or restores Ca^2+^ release via RyR1 (perhaps by facilitating its refolding). Treatment of mice with 4PBA reduced both ROS production in FDB fibres (Fig. 7a) and oxidized proteins in muscle homogenates of IT mice (Fig. 7b,c). However, 4PBA did not ‘fix' the IT associated decrease in SR Ca^2+^ release via RyR1 (Fig. 7d), suggesting that the 4PBA is not just functioning to refold RyR1 with the IT mutation.

### 4PBA improves muscle function in mice with the IT mutation

Clinically, the most important question is whether the treatment of the IT mice with 4PBA improves muscle function and increases muscle fibre size. In 10-week-old mice 4PBA significantly improved wheel running compared to WT mice (Fig. 8a). In 30-week-old mice, 4PBA treatment dramatically improved wire-hang performance in the IT mice (Fig. 8b,c), fully restoring IT function to WT levels. Maximal force generation in the soleus was decreased in muscle isolated from the 30-week-old IT mice compared to their WT littermate controls, but after 4PBA treatment, the muscle force of the IT mice significantly improved and force generation was similar to the muscle of WT mice (Fig. 8d). Serial muscle cross-sections (stained with H&E, NADH tetrazolium reductase and MHC antibodies) from the 29-week-old IT mice, their WT littermates, and both IT and WT mice treated with 4PBA for 2–3 weeks are shown in Supplementary Fig. 7. If ER stress-driven changes in protein synthesis and protein turnover underlie the decreased fibre size in the IT mice, then chronic treatment with 4PBA should restore fibre size of IT mice to a WT level. 4PBA produced an impressive increase in fibre size in all muscles tested (Fig. 8e; Supplementary Figs 2n; 3n). The effect of 4PBA on the CSA distributions in the soleus (29-week-old mice) of IT mice is shown in Supplementary Fig. 8.

We also tested the effect of 4PBA on wire hang performance in old (50 week) IT mice and again found significant improvement (Fig. 8f). 4PBA also increased fibre size in 50-week-old mice (Fig. 8g). We did not detect major ultrastructural defects in IT muscle either with or without 4PBA (Fig. 8h), but 4PBA reduced ER stress markers even in these older mice (Fig. 8i,j) and increased mitochondrial protein content (Fig. 8k,l).

## Discussion

Defining the mechanisms underlying the pathology of RyR1 myopathies is a critical first step towards developing interventions. We explored the mechanisms by which the I4895T mutation, associated with a severe myopathy in humans (I4898T), alters muscle function in mice. In marked contrast to our previous findings in mice with a RyR1 Ca^2+^ leaky mutation (Y522S in humans, Y524S in mice)^5^, the IT mutation decreases voltage-gated Ca^2+^ release and resting cytosolic Ca^2+^ levels but increases ER stress/UPR, enhances Ca^2+^ uptake/ROS production by interfibrillar mitochondria, activates proapoptotic pathways and decreases protein synthesis. Since persistent ER stress/UPR decreases protein synthesis, strengthens the contact between the ER and mitochondria, increases mitochondrial Ca^2+^ uptake and ROS production^34^, induces mPTP and drives apoptosis^40^, our findings suggested the possibility that the IT myopathy arises from a persistent elevation in ER stress/UPR. Indeed, we found ER stress/UPR markers were persistently elevated in the muscle of IT mice at different ages.

A critical question is how the IT mutation in RyR1 drives ER stress/UPR. The IT mutation in RyR1 is in close proximity to the amino acids (D4878, D4907 and E4908) involved in the binding of triadin to RyR1 (ref. 30). We found a decrease in triadin and a mislocalization of CSQ in muscle from the IT mice. CSQ polymerization and its interactions with triadin and junctin play a critical role in its localization to the junctional SR where it is in close contact with RyR1 (refs 30, 31, 32). Both triadin deficiency and CSQ mislocalization are associated with ER stress in cardiac muscle^41^,^42^. Hence, we propose that alterations in lumenal SR proteins (triadin and CSQ) occurring as a result of alterations in their interactions with IT mutated RyR1 drive ER stress/UPR in skeletal muscle.

Another consequence of the IT mutation was increased ROS production, which was blocked by Xestospongin C and RU360 (blockers of IP3R and the MCU, respectively), indicating that increased mitochondrial Ca^2+^ uptake is driving increased ROS production. Since this is occurring under conditions with reduced cytoplasmic Ca^2+^ levels, the increased mitochondrial Ca^2+^ uptake is likely to be occurring at the highly specialized microdomains of the ER/SR-MaMs. Consistent with this possibility unresolved ER stress has been shown to increase mitochondrial Ca^2+^ uptake^34^. In addition, Csordas *et al*.^48^ found that the spacing between the ER and mitochondria modulates mitochondrial Ca^2+^ uptake. Shortened distances between the ER and mitochondrial membranes are associated with increased Ca^2+^ uptake by mitochondria at ER-MaMs and mPTP^49^,^50^. The observed decrease in the length of the tethers (tightening of tethers) between the SR and interfibrillar mitochondria in IT fibres could contribute to the increased mitochondrial Ca^2+^ uptake/ROS production, but additional studies of the structure/number of ER-SR-MaMs and direct measurement of mitochondrial Ca^2+^ uptake are required. Alterations in mitochondrial function could contribute to the metabolic changes detected in the IT mice (increased weight gain on a high fat diet).

Muscle atrophy is a serious problem in most RyR1 myopathies^15^,^51^,^52^. The CSA of Type I and IIa fibres in the IT mice are decreased at least in part due to decreased muscle protein synthesis. Reduced protein synthesis is likely to result from ER stress-driven reductions in mTORC1 activity^44^. Increased REDD1 (secondary to elevated p53)^53^, decreased phosphorylation of Akt at S473 by mTORC2, and reduced translational initiation due to the increased phosphorylated eIF2 are likely to contribute to decreased mTORC1 activity.

The finding of persistent ER stress/UPR in the muscle of the IT mice led us to test the effects of a chemical chaperone, 4PBA, for ability to improve muscle function in the IT mice. 4PBA reduces markers of ER stress/UPR, but also increases protein synthesis and muscle mitochondrial protein content and reduces oxidative stress and proapoptotic markers, suggesting that mitochondrial damage and elevation of proapoptotic pathways are secondary to the ER stress/UPR. 4PBA, however, did not restore the amplitude of SR Ca^2+^ release via RyR1, suggesting that reduced Ca^2+^ release is not the primary driver of the myopathy. 4PBA is an FDA-approved drug that facilitates protein folding, in turn, suppressing ER stress-mediated apoptosis by inhibiting eukaryotic initiation factor 2α (eIF2α) phosphorylation, CCAAT (highly conserved promoter region of the Grp genes)/enhancer-binding protein homologous protein (CHOP) induction, and caspase-12 activation^54^. 4PBA also increases gene expression of antioxidants to decrease oxidative stress and protein aggregation in Parkinson disease cell model^55^. In our study, 4PBA dramatically improves muscle function and increases muscle fibre size in the IT mice at all ages tested, strongly supporting our hypothesis that this RyR1 myopathy is an ER stress/UPR disorder.

**Figure 9:**
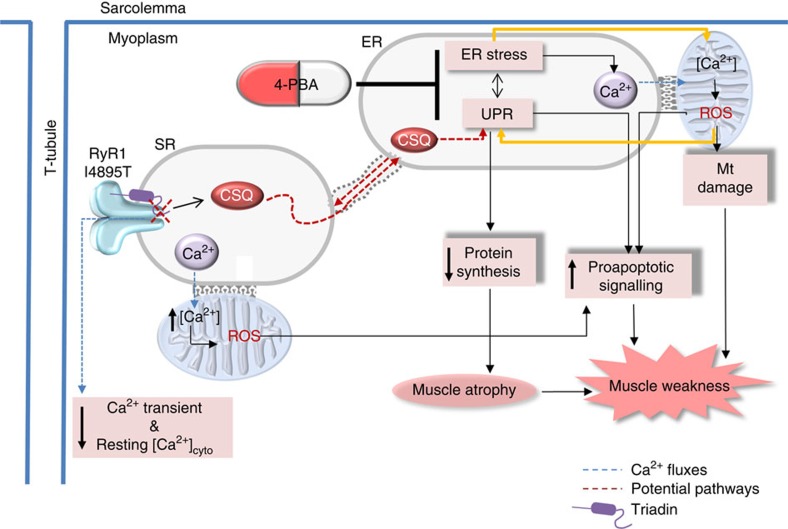
Model for effects of the IT mutation and 4PBA. The RyR1 IT mutation alters the interactions of RyR1 with SR lumenal proteins to drive ER stress/UPR, leading to decreased protein synthesis, increased mitochondrial Ca^2+^ uptake and ROS production (which could feed forward to further increase ER stress), mitochondrial damage and activation of proapoptotic pathways. 4PBA reverses pathological signalling pathways and improves muscle functions in IT mice.

In summary, our data demonstrate that persistent ER stress/UPR, decreased protein synthesis, mitochondrial ROS production/damage and elevation of proapoptotic markers are defining features of RyR1 myopathy associated with the I4895T mutation in mice, making this myopathy distinct from that of the RyR1 myopathies that arise from Ca^2+^ leak (model in Fig. 9). These findings emphasize the need to tailor therapeutic approaches for RyR1 myopathies to the mutation and emphasize the importance of distinguishing between RyR1 mutations that cause Ca^2+^ leak from those that cause persistent ER stress/UPR. It will also be important to determine if ER stress/UPR contributes in any way to the muscle weakness associated with Ca^2+^ leaky mutations in RyR1. Drugs such as rycals show promise for the Ca^2+^ leaky mutations^6^, while chemical chaperones and ER stress inhibitors may be better suited for mutations in RyR1 that produce ER stress/UPR. The sequence around amino acid I4898 in RyR1 is a hot spot for mutations that produce myopathies^9^ and these myopathies should all be evaluated for ER stress/UPR. Whether ER stress/UPR contributes RyR1 myopathies arising from RyR1 mutations at other locations in the RyR1 structure also requires further investigation. While more work is needed to confirm that this persistent ER stress is a defining component of the human RyR1 myopathies and to determine if the potential benefits of 4PBA outweigh any risks in treating this unique subclass of RyR1 myopathy, our work raises the possibility that the clinically approved drug, 4PBA, could be repurposed for use in humans with RyR1 myopathies arising from ER stress.

## Methods

### Generation of *RyR1* ^*I4895T/WT*^ knock-in mice

A genomic clone containing exons 14–23 of mice *Ryr1* was isolated from a 129/SvJ λKO-1 library. A 4.9 Kb SalI-SalI RyR1 fragment was cloned into pBluescript II KS+ (pBS, Stratagene) to generate the plasmid pMGC1. The pMGC1 was used for site-directed mutagenesis (QuickChange XL, Stratagene) to introduce both the I4898T (I4895T in mice) mutation and a new BamHI restriction site. The Sal I-Sal I region containing the I4898T mutation was completely sequenced and subcloned back into the RyR1 genomic clone. In the process of screening the 129/SvJ λKO-1 library, a tetracycline resistance gene (TetR derived from the pKOEZ-50 plasmid provided by P. Zhang) flanked by two NotI restriction sites was introduced into intron 17 of the RyR1 genomic clone. The TetR was then cut out with NotI and replaced with a lox P flanked NeoR gene expressed from the phosphoglycerate kinase promoter (PGK-NeoR) and a TetR cassette to obtain the final targeting vector (*Ryr1*^*I4898T Neo*^). The targeting vector was linearized with PmeI and electroporated into AB2.2 129 Sv/J ES cells. DNA was isolated from G418 and gancyclovir double resistant clones and subjected to Southern blot analysis. Correctly targeted ES cells were further verified for the presence of the mutation by PCR followed by restriction digestion with BamHI and direct sequencing. One correctly targeted ES clone carrying the I4898T mutation was injected into C57BL/6 blastocysts, and resulting chimeras were mated with C57BL/6 mice. The clone gave rise to germline transmission resulting F1 *Ryr1*^*I4895Tneo/+*^ mice. The *Ryr1*^*I4898Tneo/+*^ mice were mated onto Tg (EIIA-Cre) mice (a gift from Dr Graeme Mardon, Baylor College of Medicine, Houston, TX) to remove the floxed PGK-NeoR/TetR cassette, thus generating heterozygous (Ht) *Ryr1*^*I4898T/wt*^ mice. *Ryr1*^*I4898T/wt*^ mice were backcrossed on C57BL/6 background. Experimental protocols for animal research were approved by Institutional Animal Care and Use Committees (IACUC) at Baylor College of Medicine (animal protocol AN2656). Male mice were used in the current studies. Ongoing studies are assessing the phenotype and effect of 4PBA in the female mice. Generation of the IT mice is outlined in Supplementary Fig. 1.

### Materials

N-benzyl-p-toluene sulfonamide (BTS) was purchased from Tocris Biosciences and 4-chloro-m-cresol (4CmC) from PFALTZ & BAUER INC. The calcium dyes fura-2 AM and mag-fluo4 AM and the mitochondrial ROS sensor, MitoSOX Red were purchased from ThermoFisher. Xestospongin C was obtained from Abcam. RU-360 was purchased from Calbiochem. 4-phenylbutyric acid (4PBA) and all other chemicals were purchased from Sigma-Aldrich unless otherwise stated. Acrylamide solution (30%), protease inhibitor cocktail and molecular weight marker for western blotting were purchased from GenDEPOT. All antibodies used are listed in Supplementary Table 1.

### Fibretyping with cryosections and immunostaining

Muscles (soleus, EDL and diaphragm) were dissected, embedded in OCT compound (Tissue-Tek Cryomold (SAKURA)), and frozen in precooled 2-methylbutane. Frozen muscles were sectioned at 9 μm thickness using a cryostat microtome (SHANDON Cryotome E, Thermo Electron Corporation). For fibre type staining, sections were rehydrated with PBS for 10 min and incubated at 4 °C overnight with anti-MHCI (DSHB, BA-F8, IgG_2b_,1:50 dilution) and anti-MHCIIa (DSHB, SC-71, IgG_1_, 1:50 dilution) antibodies (see Supplementary Table 1 for details)^56^. Sections were washed twice in PBS and incubated at room temperature for 90 min AlexaFluor-594-conjugated goat anti-mouse IgG_2b_ and AlexaFluor-488-conjuated goat anti-mouse IgG_1_ (Life Technologies) secondary antibodies diluted 1:200 in PBS. After washing with PBS, muscle sections were mounted with Fluoromount-G mounting media (SouthernBiotech) supplemented with DAPI for nuclear staining. Imaging was performed under a fluorescence microscope (Olympus). Fibre type number was quantified and normalized by the total number of muscle fibres per field.

### Fibre cross-sectional area

Fibre type images were imported into Photoshop CSE v. 10.0 (Adobe Systems, San Jose, CA) for analysis. After establishing the measurement scale by tracing a scale bar in each image (μm) CSA of muscle fibres was measured by tracing the external border of individual muscle fibre using the Magnetic Lasso tool^56^. For accurate analysis muscle fibres exhibiting evidence of damage or processing artefacts were excluded from the analysis. Recorded values were exported into a spreadsheet program (Excel, Microsoft Inc.) for analysis and CSA values were described as μm^2^.

### Wire hang test

A wire hang test^57^ was employed to assess whole-body muscle strength and endurance. Mice were suspended by the forelimbs at the centre of a metallic wire (1 mm in diameter, 65 cm in length), which is elevated at 40 cm above the surface level. As the primary outcome, the holding impulse (g*s) is calculated as the product of the body mass (g) and the longest suspension time (s) during the 3-min test period. These studies were blinded to testers and the mice being analysed were randomized.

### Mouse activity

Briefly, to assess the food intake and cage activity, mice were placed in the Comprehensive Laboratory Animal Monitoring System (CLAM System, Columbus Instruments) equipped with running wheel at room temperature and monitored for up to 2 weeks before and after drug treatment^58^.

### Muscle force frequency and fatigue

Strips of diaphragm muscle and intact soleus and EDL muscles were removed and immersed in Kreb's ringer solution (137 mM NaCl, 5 mM KCl, 1 mM NaH_2_PO_4,_ 24 mM NaHCO_3,_ 5 mM Glucose, 2 mM CaCl_2_, 1 mM MgSO_4_, pH 7.4) oxygenated with a 95/5% mixture of O_2_/CO_2_ (ref. 56). Muscles were suspended from a force transducer and anchored in a test chamber filled with Kreb's solution. After a 20-min equilibration period, muscle optimal length (*l*_o_) and optimal stimulation voltage was determined via single twitch force generation measurements applied using platinum electrodes attached to a Grass S48 stimulator and recorded within Chart5 (version 5.2) software. Force frequency measurements were obtained using frequencies from 1–300 Hz at 200 ms/train followed by a fatigue protocol performed over 5 min per muscle. The specific fatigue protocol for each muscle used was for the soleus: 15 Hz, 200 ms duration, 1 s intervals) and for EDL: 60 Hz, 200 ms duration, 1 s intervals. All experiments were performed at 35 °C.

### Immunohistochemistry

Intact FDB fibres were plated on extracellular matrix and cultured overnight at 37 °C. Fibres were fixed for 10 min in PBS containing 4% formalin and 100 μM EGTA and then washed 3 × 5 min in PBS. Fibres were then permeabilized for 30 min in 1% Triton X-100 and blocked for 1 h in blocking buffer (2% goat serum, 0.1% Triton X-100 and 0.5% BSA). Antibodies to RyR1 (Thermo Scientific MA3-925, 1:300 dilution) and CSQ (Thermo Scientific, PA1-913, 1:400 dilution) were diluted in blocking buffer and applied overnight at 4 °C. After washing 3 × 5 min in PBS, fibres were incubated with Alexa Fluor conjugated secondary antibodies for 2 h at room temperature before being mounted using VECTASHIELD mounting media. Fibres were imaged in the Optical Imaging and Vital Microscopy Core at BCM on a Zeiss LSM 780 confocal microscope using the × 60 oil objective and images were quantified in Image J.

### Intracellular Ca^2+^ handling

FDB muscles were dissected and fibres were isolated by collagenase digestion^47^. Fibres were imaged within 24 h of isolation. Fibres were loaded with 5 μM Mag-Fluo 4 AM for 30 min at room temperature followed by 30 min washout with Tyrode's solution containing: 121 mM NaCl, 5 mM KCl, 1.8 mM CaCl_2_, 500 μM MgCl_2_, 400 μM NaH_2_PO_4_, 100 μM EDTA, 5.5 mM glucose and 24 mM NaHCO_3_, pH regulated with bubbling of CO_2_/O_2_ 5/95%, supplemented with 40 μM BTS to inhibit muscle contraction. To assess the frequency dependence of Ca^2+^ release, electrical stimulation was applied using two platinum electrodes positioned longitudinally either side of the fibre at the following stimulation frequencies: 1 Hz, 20 Hz, 60 Hz, 100 Hz (250 ms train duration), separated by a 1-min rest period. Fibres were imaged using a Zeiss LSM 510 meta confocal microscope in line scan mode with a × 20 objective (EC Plan-Neofluar). One line was acquired every 1.15 ms (3.66 μs/pixel/time). Mag-fluo 4 was excited at 488 nm and emitted light was detected with a 505 nm long-pass filter. Analyses of the area under the curve and Ca^2+^ transient kinetics was made using Sigmaplot 13.

In other experiments Mag Fluo 4 and Fura 2 loaded FDB fibres were imaged with an inverted Nikon Eclipse TE-200 microscope equipped with a Lambda DG5 illumination system and a Rolera MGi-plus CCD camera (Photometrics). Mag Fluo 4 loaded FDB fibres were stimulated with 100 Hz trains (250 ms duration, every 1.5 s; 0.17 duty cycle) for 300 s and Mag Fluo 4 was excited at 500 nm and emitted light was collected continuously at >510 nm. To measure baseline cytoplasmic Ca^2+^ isolated FDB fibres were loaded with 10 μM Fura-2AM for 1 h at room temperature followed by a 30-min washout with Tyrode's solution. Fibres were excited at 340/380 nm and fluorescence emission was measured at 510 nm. Data were acquired using Metafluor (Version 7.8) and analysed in Sigmaplot 13.

### Western blotting

Briefly, muscles were lysed with bead homogenizer (Precelly 24 lysis & homogenization, Bertin technologies) in ice-cold RIPA buffer: 25 mM Tris pH 7.6, 150 mM NaCl, 1 mM Na_3_VO_4_, 10 mM NaPyroPO_4_, 10 mM β-glycerophosphate, 10 mM NaF, 1 mM PMSF, 1X protease inhibitor cocktail (Santa Cruz), 1% NP40, 1% sodium deoxycholate and 0.1% SDS. After quantification by BCA protein assay (Thermo Scientific) equal amounts of muscle homogenates were resolved by SDS-PAGE, transferred to PVDF membrane (Millipore, Billercia, United States) and probed using primary antibodies from the vendors and at the dilutions shown in Supplementary Table 1 with LI-COR IRDye secondary antibodies (LI-COR Inc, Lincoln, United States). Immunoreactive bands were visualized and quantified by densitometry using the Odyssey Infrared Imaging System and software (LI-COR). To control for variations among different western blots and allow for multiple repeats, the absorbance intensity for each band on the western blot was normalized to the absorbance intensity of corresponding band from the control mice from the same western blot, averaged with values obtained from other western blots and plotted as % control. Some blots had multiple controls and the absorbance of the specific band was normalized to the average^56^. Full length western blots are show in Supplementary Fig. 9.

### Transmission electron microscopy for ultrastructure

Electron microscopy was performed by using methods modified from Boncompagni^14^. Muscle segments were postfixed in 1% OsO_4_ for 1 h at 4 °C, en block-stained in saturated aqueous uranyl acetate for 1 h and embedded in Spurr's Low Viscosity resin. Ultrathin sections (50–60 nm) were cut with an ultramicrotome Leica U7 (Leica Microsystem, Austria) using a Diatome diamond knife (Diatome Ltd. CH-2501 Biel, Switzerland). Sections were stained in saturated aqueous uranyl acetate and lead citrate solutions. Sections were imaged at × 3500.

### TUNEL and caspase activity assay

Diaphragm, EDL and soleus muscle tissues were lysed in lysis buffer (Triton X-100 0.1%, Tris HCl pH 8, 5 mM, EGTA 20 mM, EDTA 20 mM) and homogenized with polytron homogenizer (Precellys 24, Bertin Technologies). Protein concentrations were measured using Lowry assay. Cell apoptosis ELISA analysis was performed according to manufacturer's instructions after appropriate dilutions (Roche). For the TUNEL assay, frozen sections of soleus were fixed with fixation solution (4% paraformaldehyde in PBS pH 7.4) for 20 min, washed with PBS for 30 min and then incubated with permeabilization solution (0.1% Triton X-100, 0.1% sodium citrate). Fixed and permeabilized slides were treated with TUNEL reaction mixture (terminal deoxynucleotidyl transferase from calf thymus, recombinant in *E. coli* and nucleotide mixture – Roche). Nuclear stain DAPI was used to label the nuclei. For caspase activation assay, acetyl-Asp-Glu-Val-Asp-7-amido-4-methylcoumarin (Ac-DEVD-AMC) was used as substrate. Caspase activity was calculated from changes in fluorescent AMC (Excitation 360 nm, emission 460 nm).

### Oxyblot for oxidized proteins

Oxidized proteins were assessed by the OxyBlot Protein Oxidation Detection Kit (Millipore) in 10 μg of soleus, EDL and diaphragm lysates following the manufacturer's protocol.

### Mitochondrial and cytosolic fractionation

Fresh muscles were homogenized using a glass homogenizer with ice-cold mitochondrial isolation buffer (10 mM Tris–HCl pH 7.2, 320 mM sucrose, 1 mM ethylene diamine tetra acetic acid, 1 mM dithiothreitol, 1 mg ml^−1^ bovine serum albumin) and centrifuged at 1,500 g for 5 min to remove debris and nuclear fractions. Supernatant was transferred to a new tube and was further centrifuged at 15,000 g for 20 min for the phase separation of the mitochondrial pellet and cytosolic supernatant. Mitochondrial pellet was lysed in RIPA buffer (25 mM Tris pH 7.6, 150 mM NaCl, 1 mM Na_3_VO_4_, 10 mM NaPyroPO_4_, 10 mM β-glycerophosphate, 10 mM NaF, 1 mM PMSF, 1X protease inhibitor cocktail (Santa Cruz), 1% NP40, 1% sodium deoxycholate, and 0.1% SDS (reagents from Sigma Aldrich) and protein concentrations of each fraction were determined by the BCA Protein Assay (Thermo Scientific), according to the manufacturer's protocol.

### XBP1 splicing

Total RNA was extracted from mouse tissue using TRIzol reagent (Life Technologies), and reverse transcribed into cDNA using iScript cDNA Synthesis Kit (BIO-RAD). Quantitative real-time polymerase chain reaction (qRT-PCR) was performed with iQ SYBR Green Supermix (BIO-RAD) using primer sets specific for target and reference genes (PrimerBank, MGH). The relative splicing level of Xbp1 was determined by normalizing the expression of spliced Xbp1(S) to unspliced Xbp1(U) mRNA. The relative transcription levels of all other genes were determined by normalizing the expression of target genes to the reference 18 s rRNA. Relative gene expression was calculated based on the reaction cycle threshold (Ct) values using Pfaff method^59^. Primers: Xbp1s forward 5′-GAGTCCGCAGCAGGTG-3′ and reverse 5′-GTGTCAGAGTCCATGGGA-3′; Xbp1u forward 5′-GACTATGTGCACCTCTGCAG-3′ and reverse 5′-CTGGGAGTTCCTCCAGACTA-3′.

### Mitochondrial ROS

Mitochondrial ROS production was measured using methods modified from Cheng *et al*.^60^ and Aydin *et al*.^61^. Briefly, single FDB fibres were incubated at room temperature with 5 μM MitoSOX Red in Tyrodes solution for 15 min, followed by a 45-min washout period in indicator-free Tyrodes solution. MitoSOX loaded fibres were imaged on a Zeiss LSM 510 meta laser scanning confocal microscope using a × 20 objective. Fibres were excited with an argon laser at 488 nm and emission was collected using a 560LP filter. XY images of baseline mitoSOX fluorescence were always captured within 20 min of completion of mitoSOX loading. Antimycin A (20 μM) was added to the chamber at the end of each experiment to verify the rise in MitoSOX fluorescence was mitochondrial. To determine the effect of blocking mitochondrial Ca^2+^ uptake or IP_3_R mediated Ca^2+^ release on mitochondrial ROS production, in some experiments fibres were incubated for 1 h with 10 μM RU-360, or with 1 μM Xestospongin C for 20 min before MitoSOX loading.

### SUnSET assay for detection of puromycin-labelled proteins

For assessment of newly synthesized proteins we used *in vivo* puromycin labelling^46^,^62^. Briefly, mice (14–15 weeks of age) were deprived of food for 6 h and refed for 2 h. Propofol (18 μl g^−1^) was intraperitoneally injected in mice after 70 min of refeeding. After 10 min, mice were intraperitoneally injected with puromycin (0.04 μmol g^−1^ body weight) and killed 35 min later. Excised muscles were processed for western blotting with anti-puromycin antibody (KeraFAST EQ-001, 1:1,000 dilution). Puromycin-labelled proteins were normalized to total proteins of the same blot stained with the Swift Membrane Stain kit (G-Biosciences).

### Chronic treatment with 4BPA

Age-matched IT and WT littermates were used for chronic (up to 6 weeks) treatment. For chronic treatment, 4PBA (Sigma) was dissolved at a concentration of 200 mg dl ^−1^and treated ad-libitum. Approximate water consumption was 3–5 ml per day per mouse. Therefore, each mouse consumes about 6–10 mg per day per mouse.

### Aggresome detection

All procedures were performed as described in the manufacturer's protocol (Enzo Life Sciences). Briefly, muscle frozen sections from soleus and EDL were placed at room temperature for 10 min and rehydrated in 1X PBS. After removing excess buffer dual detection reagent (1 μl of ProteoStat Aggresome detection reagent and 2 μl of Hoechst 33342 in 200 μl of 1X assay buffer) was added to sections and incubated for 2 h at room temperature. After washing with 1X PBS, slides were mounted with coverslip using Fluoromount-G (SouthernBiotech). Imaging was performed under × 20 magnification through an Olympus DP70 camera (Olympus America, Center Valley, PA). Fluorescence intensity of each image was quantified using Image-J software.

### Statistical analyses

Differences between means of two groups were analysed for significance using two tailed Student's *t*-tests. Data are also subjected to a normality test. If data fail the normality test they are then subjected to Mann–Whitney rank sum test. Only those data with *P<0.05, **P<0.01 or ***P<0.001 are considered significant. Outliers, defined as those values that were greater than 2 standard deviations away from the mean, were removed.

### Data availability

The data that support the findings of this study are available from the corresponding author upon reasonable request.

## Additional information

**How to cite this article:** Lee, C. S. *et al*. A chemical chaperone improves muscle function in mice with a RyR1 mutation. *Nat. Commun.* **8,** 14659 doi: 10.1038/ncomms14659 (2017).

**Publisher's note:** Springer Nature remains neutral with regard to jurisdictional claims in published maps and institutional affiliations.

## Supplementary Material

Supplementary Information Supplementary Figures and Supplementary Tables.
