# Supplementary material for: A chemical chaperone improves muscle function in mice with a RyR1 mutation
Source: Nat Commun. 2017 Mar 24;8:14659. doi: 10.1038/ncomms14659 (PMC5376670; doi:10.1038/ncomms14659)
Supplement: Supplementary Information — Supplementary Figures and Supplementary Tables. [file ncomms14659-s1.pdf]

Supplementary Materials

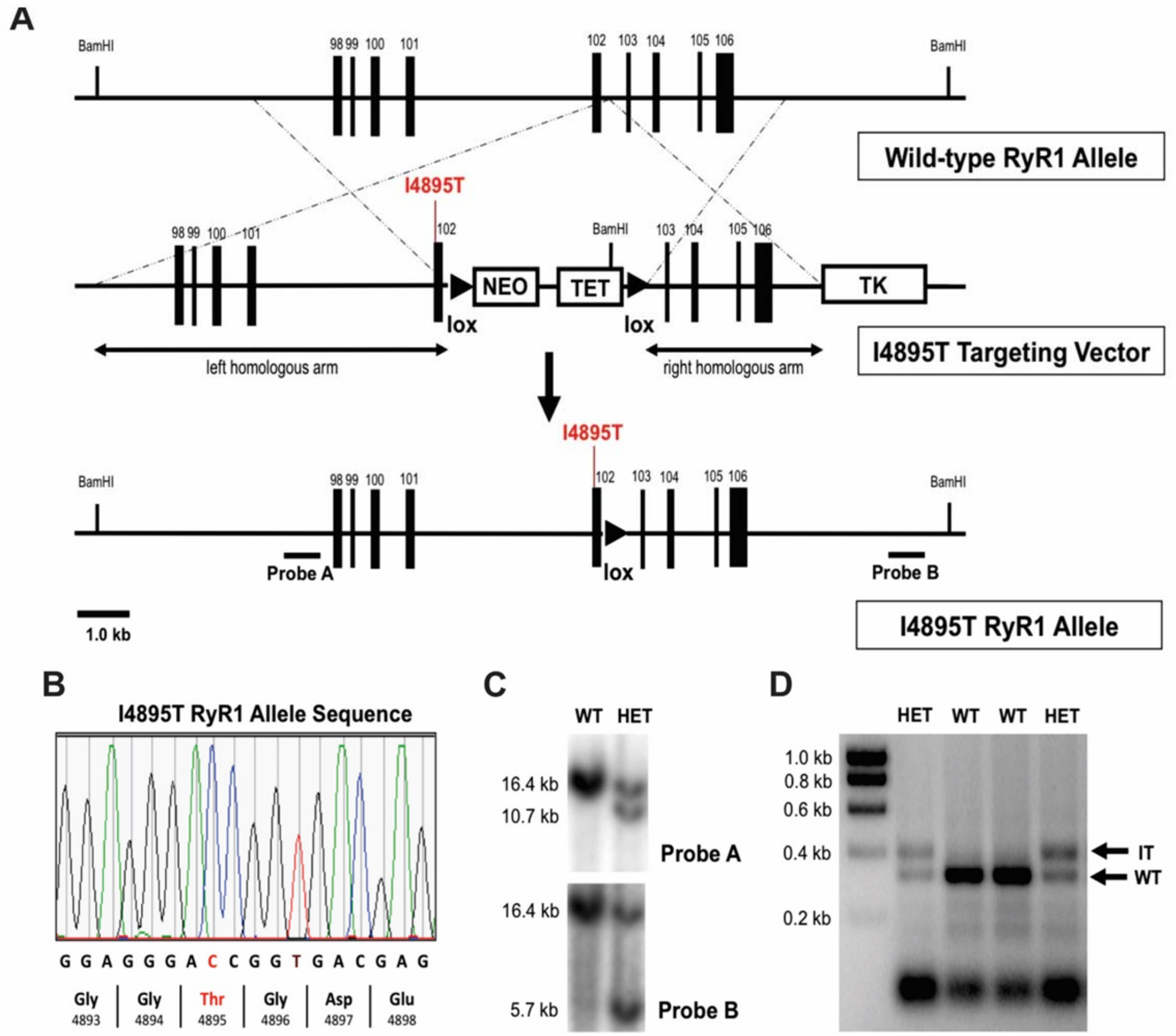

Supplementary Fig. 1. Creation of I4895T mice. A. Generation and verification of RyR1 I4895T mice. Targeted I4895T knockin allele after removing NEO-TET cassette is shown on the bottom of A. **B.** Sequencing of I4895T targeting construct. **C.** Genotyping of I4895T by Southern blot. **D.** Genotype differentiation of I4895T and WT by PCR.

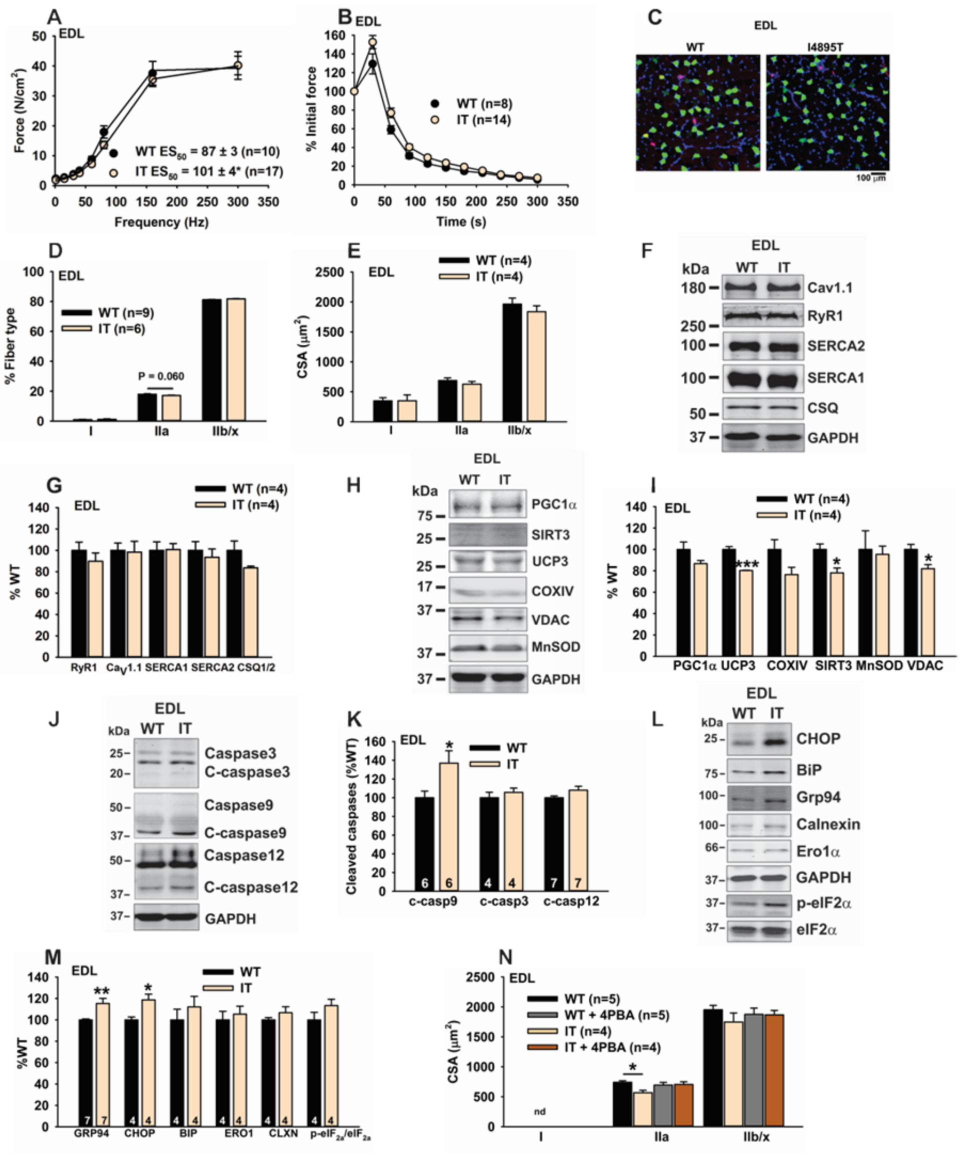

Supplementary Fig. 2. Effect of the IT mutation on the EDL muscle. A. Force frequency curve. **B.** Fatigue curve. **C.** Representative fiber type immunofluorescence images of 8-week-old IT and WT mice. **D.** Analysis of fiber types. **E.** Analysis of CSA. **F.** Representative western blot images for Cav1.1, RyR1, SERCA1/2, CSQ, and GAPDH in EDL of IT and WT littermates. **G**. Analysis of calcium handling proteins (depicted as %WT) in EDL. **H.** Representative western blot images of mitochondrial proteins, Sirt3, UCP3, COXIV, VDAC and MnSOD in EDL of IT and WT littermates. PGC1α and GAPDH are used as a master regulator for the mitochondrial biosynthesis and as a normalizing protein, respectively. **I.** Analysis of intensity of bands in panel H. **J.** Representative western blots of cleaved caspases in the EDL of IT and WT mice. **K.** Analysis of cleaved caspases in the EDL. **L.** Representative western blot of proteins involved in ER stress in EDL of IT and WT littermates. **M.** Analysis of ER stress markers in EDL. **N.** Analysis of CSA of the EDL fibers from 29-week-old mice WT and IT mice treated with or without chronic 4PBA (up to 4 weeks). nd, not detected. Data are shown as mean ± SEM. *P<0.05, **P<0.01, ***P<0.001.

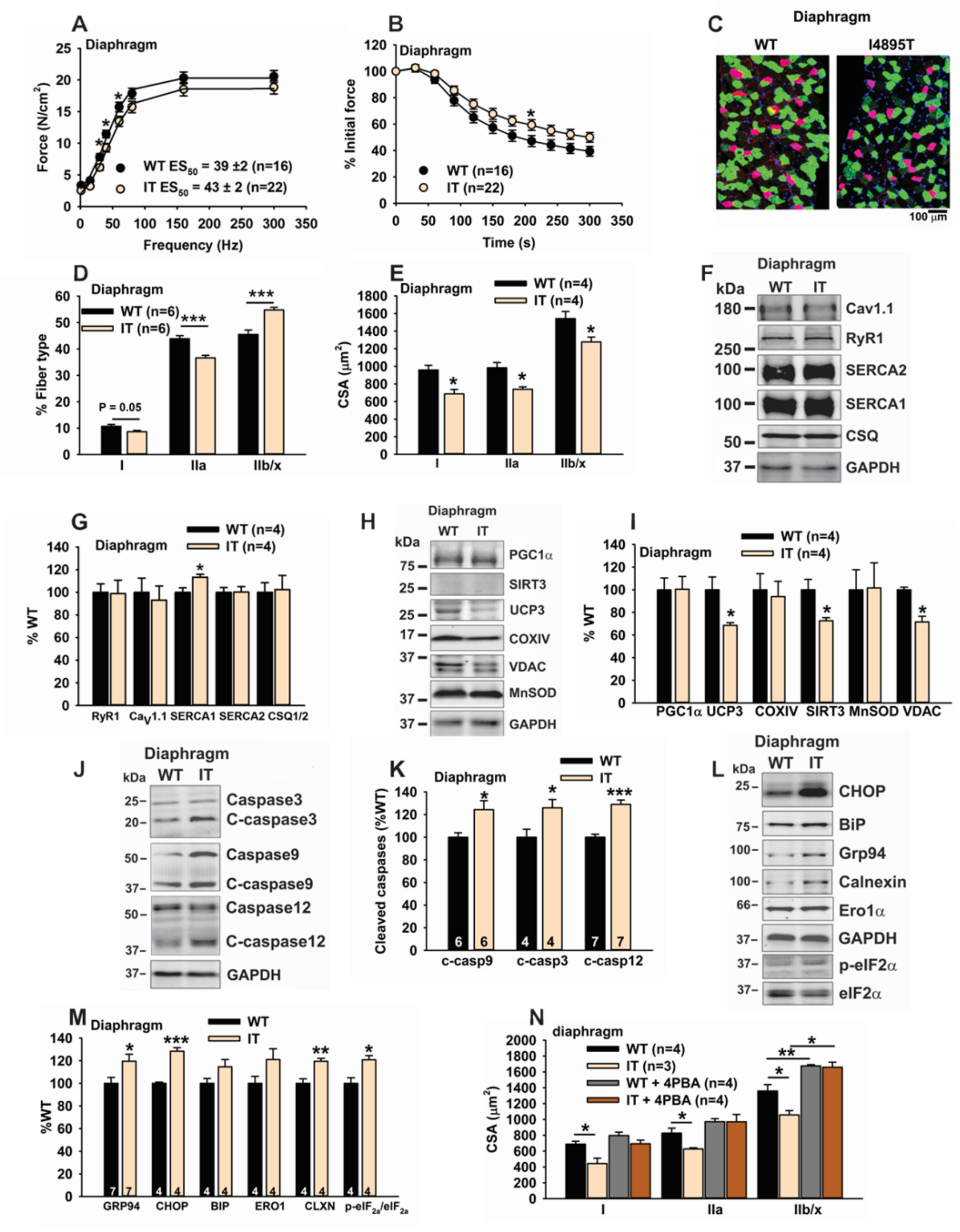

Supplementary Fig. 3 Effects of the IT mutation in the diaphragm. A. Force frequency curve of diaphragm. **B.** Fatigue curve of diaphragm. **C.** Representative fiber type immunofluorescence images of 8-week-old IT and WT mice. **D.** Analysis of fiber types. **E.** Analysis of CSA. **F.** Representative western blot images for Cav1.1, RyR1, SERCA1/2, CSQ, and GAPDH in EDL of IT and WT littermates. **G**. Analysis of calcium handling proteins (depicted as %WT) in EDL. **H.** Representative western blot images of mitochondrial proteins, Sirt3, UCP3, COXIV, VDAC and MnSOD in diaphragm of IT and WT littermates. PGC1α and GAPDH are used as a master regulator for the mitochondrial biosynthesis and as a normalizing protein, respectively. **I.** Analysis of intensity of bands in panel H. **J.** Representative western blots of cleaved caspases in the diaphragm of IT and WT mice. **K.** Analysis of cleaved caspases in the diaphragm. **L.** Representative western blot of proteins involved in ER stress in the diaphragm of IT and WT littermates. **M.** Analysis of ER stress markers in the diaphragm. **N.** Analysis of CSA of the diaphragm fibers from 29-week-old mice WT and IT mice treated with or without chronic 4PBA (up to 4 weeks). Data are shown as mean ± SEM. *P<0.05, **P<0.01, ***P<0.001.

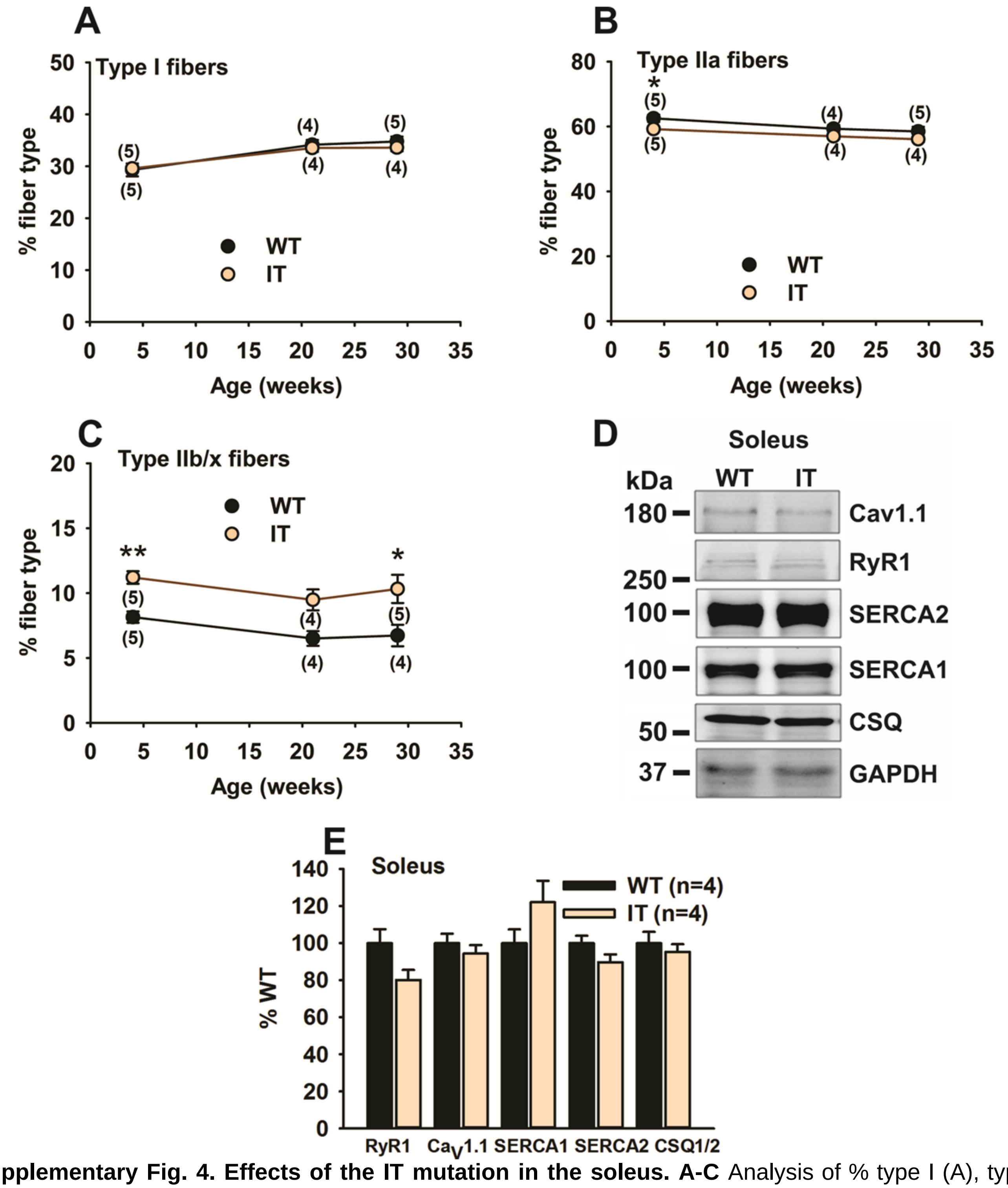

Supplementary Fig. 4. Effects of the IT mutation in the soleus. A-C Analysis of % type I (A), type IIa (B) and type IIb/x fibers (C) in the soleus of IT and WT littermate mice at 3 different age groups. Number in bracket means mouse numbers for the experiments. **D.** Representative western blot images for Cav1.1, RyR1, SERCA1/2, CSQ, and GAPDH in the soleus of IT and WT littermates. **E**. Analysis of calcium handling proteins (depicted as %WT) in the soleus. Data are shown as mean ± SEM. *P<0.05, **P<0.01.

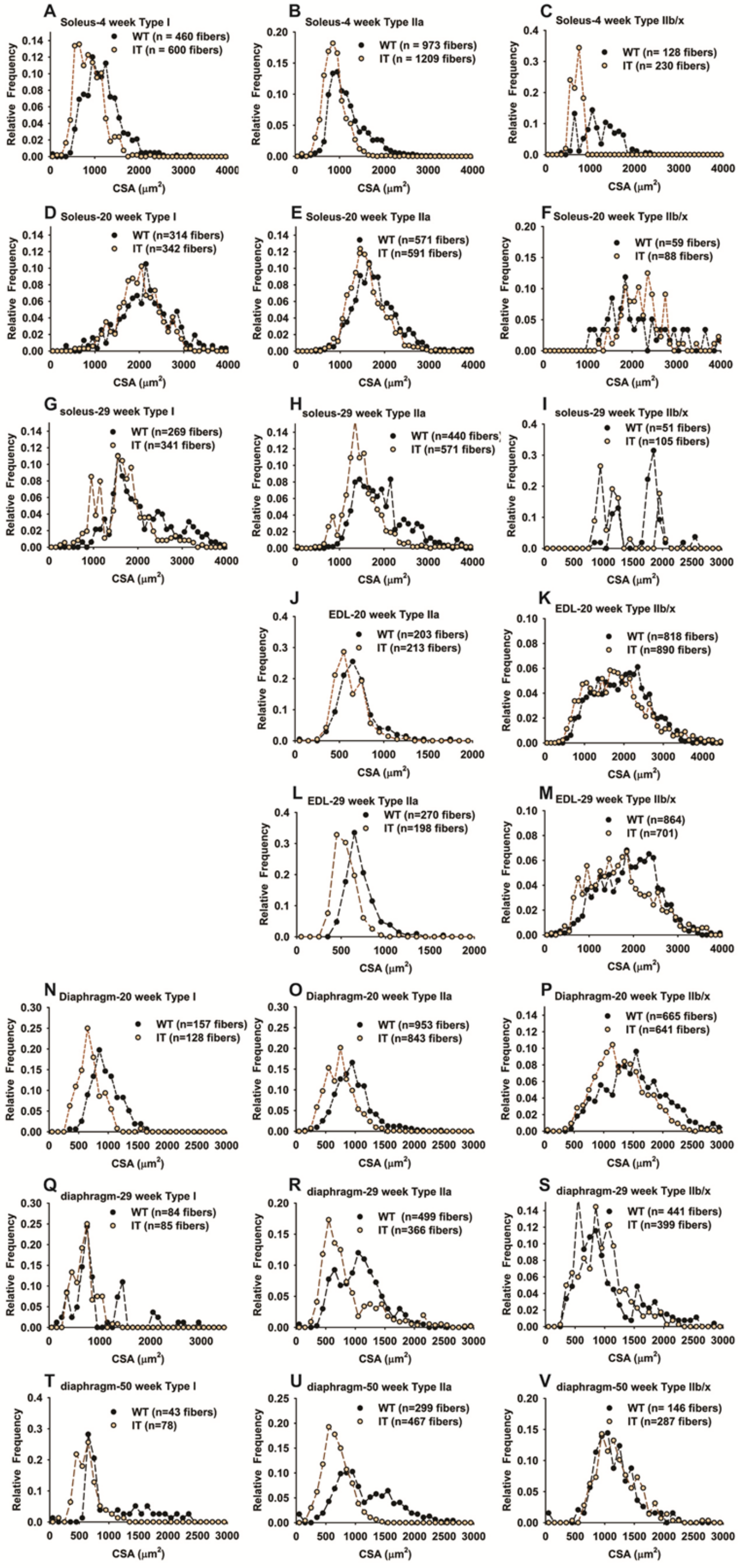

Supplementary Fig. 5. Fiber CSA Frequency Distributions. The analysis of cross-sectional area distributions in soleus (A-I), EDL (J-M) and diaphragm (N-V) from IT and WT littermate mice following developmental stages, 4 weeks (A-C), 20 weeks (D-F, J,K and N-P), 29 weeks (G-I, L,M and Q-S) and 50 weeks (T-V).

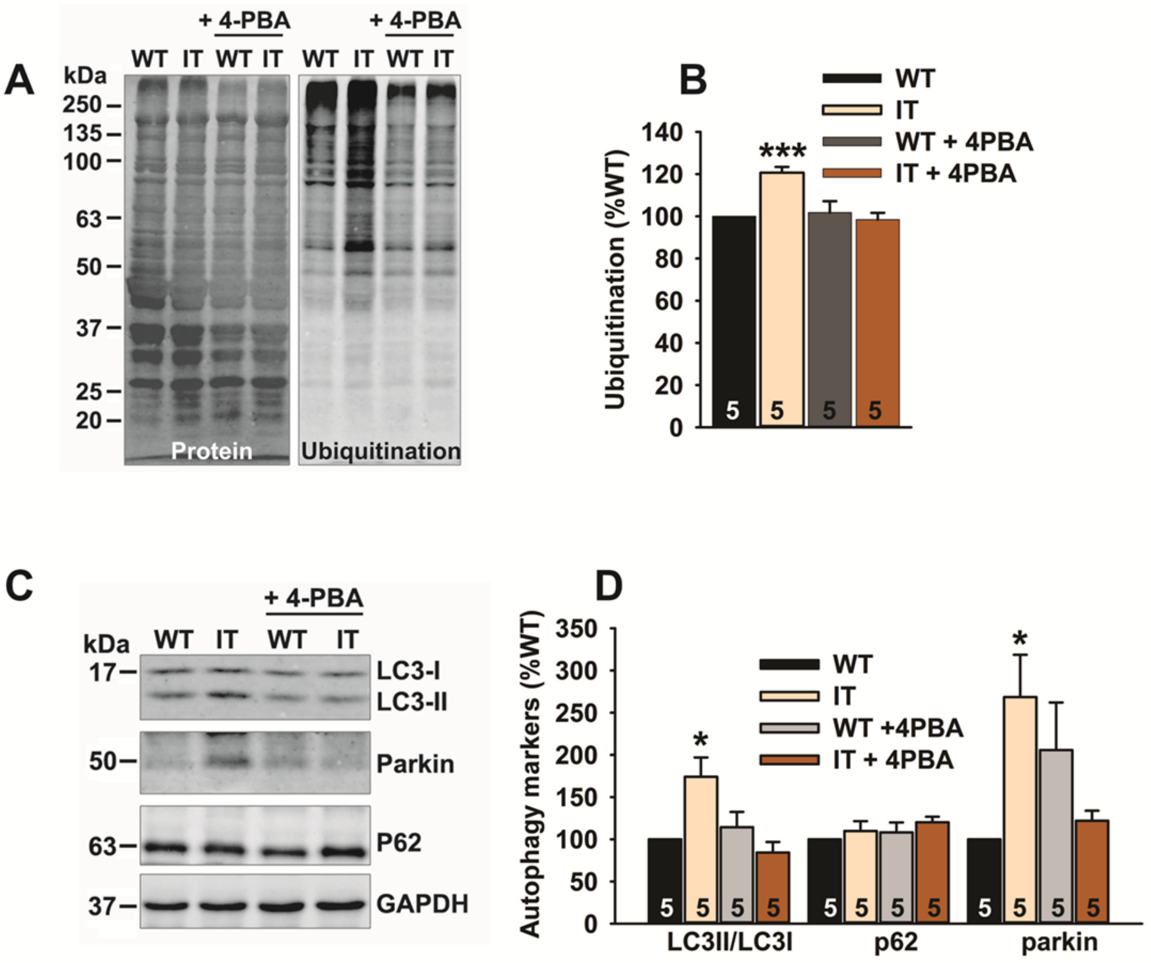

Supplementary Fig. 6. Effect of 4-PBA treatment on markers of protein degradation and autophagy. A. Representative western blot for ubiquitination of proteins in soleus muscle from IT and WT littermates without or with 4PBA for 2 weeks. **B**. Analysis of ubiquitination in panel A. **C**. Representative western blot for autophagy markers, LC3-I and –II, parkin, and p62 in lysates of soleus muscle from IT and WT littermates without or with 2 weeks 4PBA treatment. **D**. Analysis of autophagic markers shown in C. Data are represented as mean ± SEM. *p<0.05, ***p<0.001.

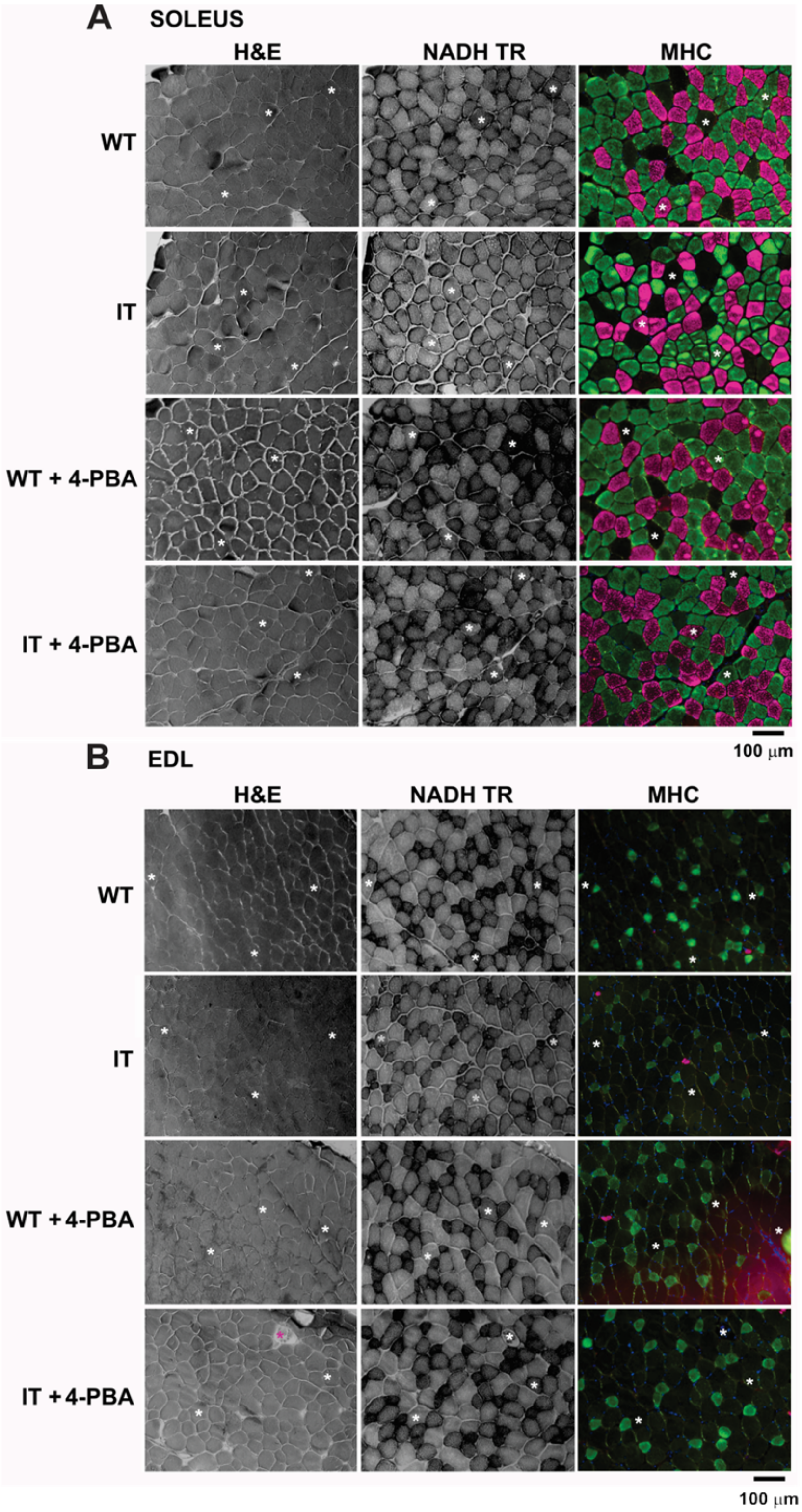

Supplementary Fig. 7. Muscle fiber staining with H&E, NADH-TZ (NADH tetrazolium for mitochondrial staining) and MHC in the soleus (A) and EDL (B) muscles of IT and WT littermates without or with chronic 4-PBA treatment (up to 3 weeks). Asterisks indicate the same fiber in each row. Bar is 100 μm.

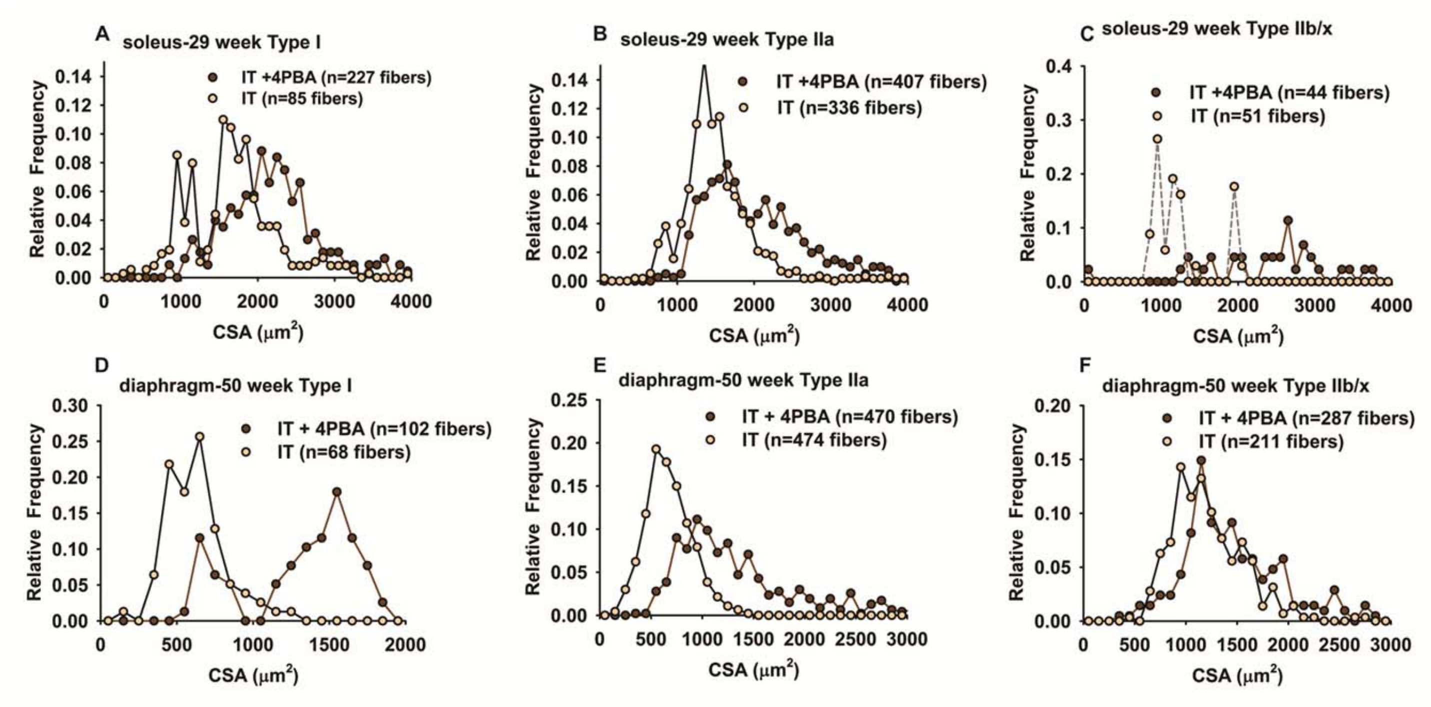

Supplementary Fig. 8. Effect of 4PBA on fiber CSA distributions. A-C. Analysis of fiber CSA distributions in the soleus of 29-week-old IT without or with chronic 4PBA treatment (up to 3 weeks). **D-F.** Analysis of fiber CSA distributions in the diaphragm of 50-week-old IT without or with chronic 4PBA treatment (up to 6 weeks). Analyzed fibers were from 3 to 4 different mice.

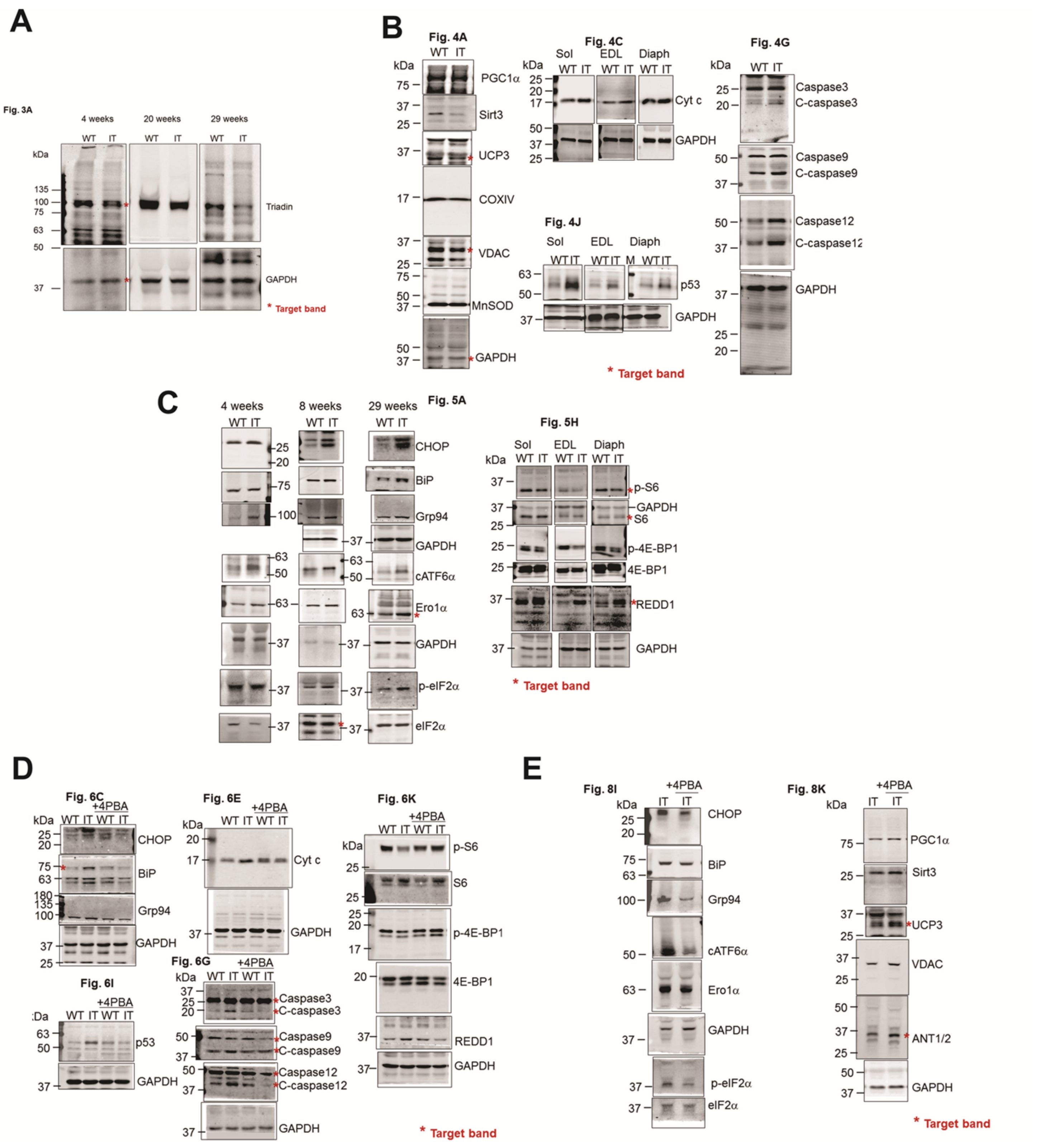

Supplementary Fig. 9 Uncropped western blots in the main figures. Blots after transferring of proteins were cut along molecular weight markers to obtain multiple blots. **A.** Uncropped images for **Fig. 3A.** Representative western blots of triadin levels in soleus muscle from WT and IT mice at different ages. **B.** Uncropped images for **Fig. 4. 4A.** Western blots for mitochondrial proteins in homogenates of soleus muscles from WT and IT mice. **4C.** Western blots of cytosolic cytochrome c in soleus from WT and IT. **4G.** western blots for caspases. **4J.** Western blot for p53 in muscles of IT and WT. **C.** Uncropped images for **Fig. 5. 5A.** Western blot of proteins involved in ER stress in the soleus of 4, 8 and 29-week-old IT and WT mice. **5H.** Western blot of proteins downstream of mTORC1 activation and REDD1. **D.** Uncropped images for **Fig. 6. 6C.** Western

blots for the effect of 2 week treatment of mice with 4PBA on ER stress markers in the soleus **6E.** Western blot for cytosolic cytochrome c in the cytosolic fraction of soleus from WT and IT mice treated with and without 4PBA for 2-3 weeks. **6G.** Western blot showing the effect of 2 week treatment of 4PBA on cleaved caspases in the soleus. **6I.** Western blots of p53 in the soleus of IT and WT littermates without or with 4PBA treatment for 2-3 weeks. **6K.** western blot of proteins downstream of mTORC1 in the soleus of IT and WT littermates without or with 4PBA treatment for 2-3 weeks. **E.** Uncropped images for **Fig. 8. 8I.** western blot of ER stress proteins in 50-week-old IT mice treated with and without 4PBA. **8K.** Western blot of mitochondrial proteins from 50-week-old IT mice with and without 4PBA treatment.

Supplementary Table 1: Antibodies used

Antibody	Company	WB	IHC	Cat. No.
RyR1	Thermo Scientific	1:2000	1:300	MA3-925
$Ca_v1.1\alpha$	Thermo Scientific	1:1000		MA3-920
Calsequestrin	Thermo Scientific	1:1000	1:400	PA1-913
SERCA1	Thermo Scientific	1:1000		MA3-911
SERCA2	Thermo Scientific	1:1000		MA3-919
Triadin	Thermo Scientific	1:1000		MA3-927
GAPDH	Santa Cruz	1:500		sc-20357
Phospho Akt1 (S473)	Cell Signaling	1:1000		#9271
Phospho Akt1 (T308)	Cell Signaling	1:1000		#2965P
Akt1/2	Santa Cruz	1:500		sc-1619
REDD1	Proteintech	1:1000		10638-1-AP
Myosin Heavy Chain 1	DSHB		1:50	BA-F8
Myosin Heavy Chain 2a	DSHB		1:50	sc-71
Myosin Heavy Chain 2b	DSHB		1:50	BF-F3
Phospho-eIF2α (Ser51)	Cell Signaling	1:1000		#3597
eIF2α	Cell Signaling	1:1000		#772
BiP/Grp78	Cell Signaling	1:1000		#3177
CHOP	Cell Signaling	1:1000		#2895
ATF6α	ENZO Life Sciences	1:1000		ALX-804-381-C100
Calnexin	Cell Signaling	1:1000		#2679
Grp94	Cell Signaling	1:1000		#2104
Ero1α	Novus Biologicals	1:1000		NB100-2525
p53	Santa Cruz	1:500		sc-6243
caspase 3	Santa Cruz	1:500		sc-7148
caspase 9	Cell Signaling	1:1000		#9504
caspase 12	Cell Signaling	1:1000		#2202
Cytochrome c	Cell Signaling	1:1000		#4280
ANT1/2	Santa Cruz	1:500		sc-9299
Sirt3	Cell Signaling	1:1000		#2627
UCP3	abcam	1:1000		ab3477
COXIV	Cell Signaling	1:1000		#4844
MnSOD	Santa Cruz	1:500		sc-133254
PGC1α	Santa Cruz	1:500		sc-13067
VDAC	Cell Signaling	1:1000		#4866
LC3	MBL International	1:2000		PD014
Parkin	Cell Signaling	1:1000		#4211
p-62	Proteintech	1:1000		18420-1-AP
Phospho-S6 (S235/236)	Cell Signaling	1:1000		#4858
S6	Cell Signaling	1:1000		#2317
Phospho-4E-BP1 (T37/47)	Cell Signaling	1:1000		#2855
4E-BP1	Cell Signaling	1:1000		#9644
Puromycin	KeraFAST	1:1000		EQ-0001
Ubiquitin	Santa Cruz	1:500		sc-8017

WB=Western blotting, IHC=Immunohistochemistry

Abcam (Cambridge, MA, United States); Cell Signaling (Danvers, MA, United States); DSHB (Iowa City, United States); KeraFAST (Boston, MA, United States); Millipore (Billerica, Massachusetts, United States); ProteinTech group (Chicago, IL, United States); Santa Cruz (Santa Cruz, CA, United States); Thermo Scientific (Waltham, MA, United States); ENZO Life Sciences (Farmingdale, NY, United States); MBL (Woburn, MA, United States); Novus Biologicals (Littleton, CO, United States).
